# Direct Photon-by-Photon Analysis of Time-Resolved Pulsed Excitation Data using Bayesian Nonparametrics

**DOI:** 10.1016/j.xcrp.2020.100234

**Published:** 2020-10-14

**Authors:** Meysam Tavakoli, Sina Jazani, Ioannis Sgouralis, Wooseok Heo, Kunihiko Ishii, Tahei Tahara, Steve Pressé

**Affiliations:** 1 Department of Physics, Indiana University-Purdue University, Indianapolis, IN 46202, USA; 2 Center for Biological Physics, Department of Physics, Arizona State University, Tempe, AZ 85287, USA; 3 Molecular Spectroscopy Laboratory, RIKEN, 2-1 Hirosawa, Wako, Saitama 351-0198, Japan; 4 Ultrafast Spectroscopy Research Team, RIKEN Center for Advanced Photonics (RAP), 2-1 Hirosawa, Wako, Saitama 351-0198, Japan; 5 School of Molecular Sciences, Arizona State University, Tempe, AZ 85287, USA; 6 Lead Contact

## Abstract

Lifetimes of chemical species are typically estimated by either fitting time-correlated single-photon counting (TCSPC) histograms or phasor analysis from time-resolved photon arrivals. While both methods yield lifetimes in a computationally efficient manner, their performance is limited by choices made on the number of distinct chemical species contributing photons. However, the number of species is encoded in the photon arrival times collected for each illuminated spot and need not be set by hand *a priori*. Here, we propose a direct photon-by-photon analysis of data drawn from pulsed excitation experiments to infer, simultaneously and self-consistently, the number of species and their associated lifetimes from a few thousand photons. We do so by leveraging new mathematical tools within the Bayesian nonparametric. We benchmark our method for both simulated and experimental data for 1–4 species.

## Graphical abstract

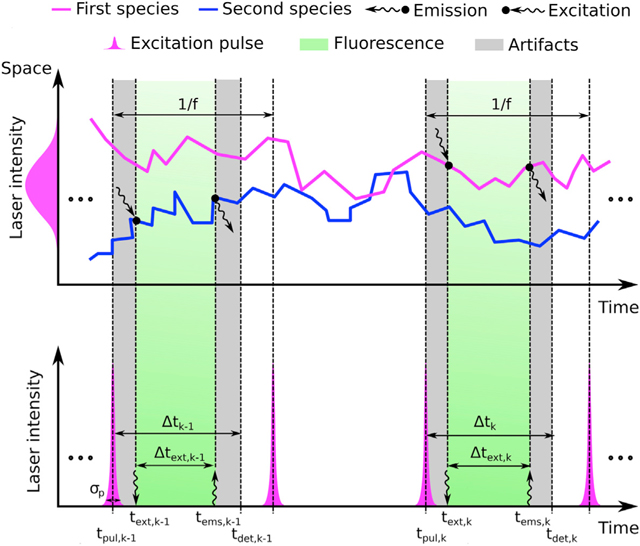

Here, Tavakoli et al. propose the direct photon-by-photon analysis of data drawn from pulsed excitation experiments to infer, simultaneously and self-consistently, the number of species and their associated lifetimes from as little as a few thousand photons. This is achieved by leveraging new mathematical tools within the Bayesian nonparametric (BNP) paradigm.

## INTRODUCTION

Fluorescence microscopy provides a means to selectively monitor the dynamics and chemical properties of fluorophores or labeled molecules.^1–13^ In this study, our focus is on methods that use pulsed illumination^14–18^ or illumination modulated at a fixed frequency^18–23^ at 1 spot. Photon arrival times assessed in these methods encode critical information on the excited state lifetime or the number of different chemical species contained in the sample under imaging. This is the basis of lifetime imaging,^13,24–27^ which has been used to reveal information on local pH,^28,29^ oxygenation,^28^ and other cellular metabolic traits^23,30^ reporting back on the breadth of cellular microenvironments.

Maximum likelihood or traditional (parametric) Bayesian methods^31–35^ are common starting points in the analysis of photon arrivals or photon arrival histograms derived from pulsed illumination (i.e., time-correlated single-photon counting [TCSPC] data).^2,36–38^

In pulsed illumination,^39,40^ photon arrival times are analyzed^41–44^ under the assumption of a known number of molecular species with unknown lifetimes to be determined^31–35,45–50^ This approach is best illustrated in discussing photon arrival histograms, which are typically fitted using multi-exponentials^49,51^ to identify the lifetime of each species. That is, lifetimes, τ_*m*_, and the weights of the *m*^*th*^ lifetime component, *a*_*m*_, are modeled and determined using multi-exponential decay fits of the form

(Equation 1) $$I(t)=\sum_{m=1}^{M}a_{m}\text{exp}\left(-\frac{t}{\tau_{m}}\right)$$

where *I*(*t*) is the intensity of photons arriving at time *t*.

In Equation 1, the number of exponential components, *M*, must be specified before the data can be used to find τ_1_, …, τ_*M*_ and *a*_1_, …, *a*_*M*_. Typically, *M* is specified according to some goodness-of-fit metric that safeguards against overfitting,^33^ as we discuss in the Supplemental Experimental Procedures. Within a maximum likelihood or parametric Bayesian paradigm, too large an *M* must be penalized according to *post hoc* criteria.^52–55^ Other methods for deducing *M* rely on pole decompositions^56^ or Laplace-Padé expansions^57^ requiring exceedingly large datasets.

Another general method of analysis of lifetime data relies on phasors.^58–62^ Phasor analysis is appropriate for data from samples illuminated by light whose intensity is modulated at a fixed frequency.^21,58,63–65^ In this case, the intensity of the light emitted by the sample is also modulated and phase shifted.^18,59^ In particular, for a modulation frequency of ω, the measurements may be used to obtain the phase shift φ and the intensity modulation ratio *m* (see Figure S10). The phase shift and intensity modulation ratio, in turn, determine 2 coordinates (*G,S*) in a “phasor plot”

(Equation 2) G(ω)=mcosϕandS(ω)=msinϕ.

Lifetime values of the photon-emitting fluorophores can then be deduced from the points on the phasor plot.^60–62^

**Figure 1.**
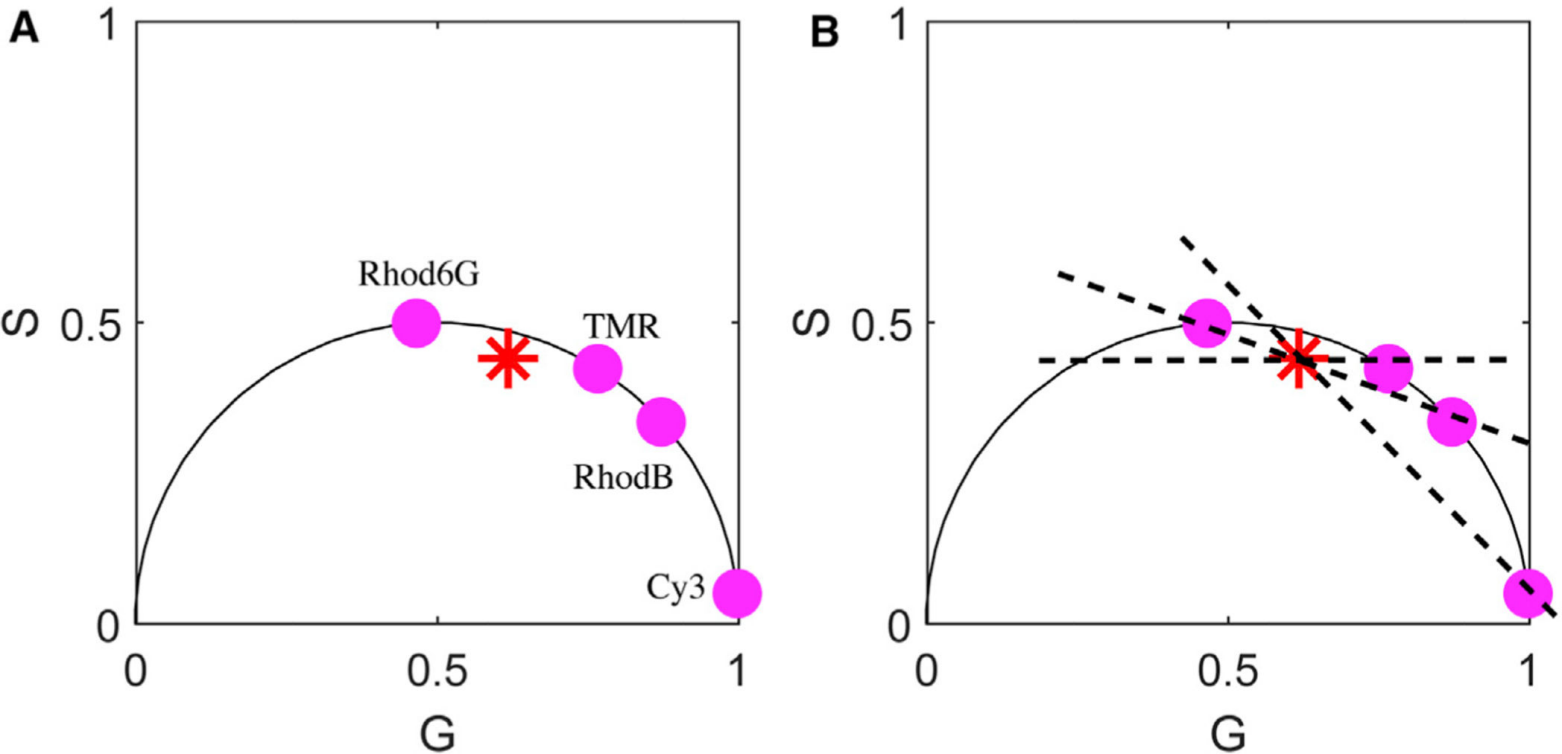
Phasor Analysis Provides the Lifetimes of Chemical Species but Not an Independent Measure of the Number of Chemical Species (A) is a typical phasor plot, as expected with a 4-component mixture. The red asterisk (Rhod-6G, TMR, RhodB, and Cy3) is subject to pulsed illumination. From this figure, it is not possible to discern the number of chemical species contributing to the phasor plot. What is more, as we can see in (B), if we assume 2 species, many choices of lifetimes could be warranted by the data as evidenced by the placement of the dashed diagonal lines. The point of intersection of these diagonal lines with the phasor plot’s hemisphere would be needed to deduce the lifetimes of a 2-component mixture if we had hypothesized this mixture to be composed of 2 species (as opposed to the correct number, 4). (B) superposes the phasor plots for each species measured independently. Their mixture is what yields the subfigure on the left, whose identity as a 4-component mixture is not apparent.

Phasor analysis is especially intuitive as it allows us to immediately deduce whether more than one lifetime component is present.^66,67^ In particular, mono-exponential lifetimes fall somewhere on the semicircle of radius 1/2 beginning at coordinate (1,0) and moving counterclockwise to (0,0) (see Figure 1). Deviations thereof imply a mixture of lifetimes. (Full details are provided in the Supplemental Experimental Procedures.) A variant of phasor analysis also holds for pulsed excitation.^60,68,69^ The advantages and drawbacks of phasor analysis are similar to those of the direct analysis of photon arrivals or histograms of photon arrivals from TCSPC data in that the number of species must be known in advance. What is more, the retrieval of lifetime information from phasor analysis requires independent knowledge of not only the number of species but, often, also the lifetimes of all but one unknown species whose lifetime is to be determined from a mixture of chemical species^27,60,70,71^ (see Figure 1).

While both approaches we have just described, direct photon analysis and phasors, yield lifetimes in a computationally efficient manner, their greatest limitation is the requirement that the number of species, *M*, be pre-specified as it otherwise cannot be learned independently, although, in principle, it is encoded in the data. However, learning the number of species is critical as it may be unknown before collecting the data for a number of reasons.^68,72–74^ At higher computational costs, we could learn not only the number of species but also full joint distributions over the possible number of species and their associated lifetimes, which are encoded in the photon arrivals. In other words, we could determine the relative probability over having 3 versus 4 species, say, not just the most probable number of species. Ideally, to allow for higher flexibility in the experimental setting, we need to achieve this with the same or fewer photon arrivals than is required in direct photon and phasor analysis to reveal the lifetimes alone. To do so, we need to relinquish the traditional (parametric) Bayesian paradigm that assumes a fixed model structure (i.e., a fixed number of species).

**Figure 2.**
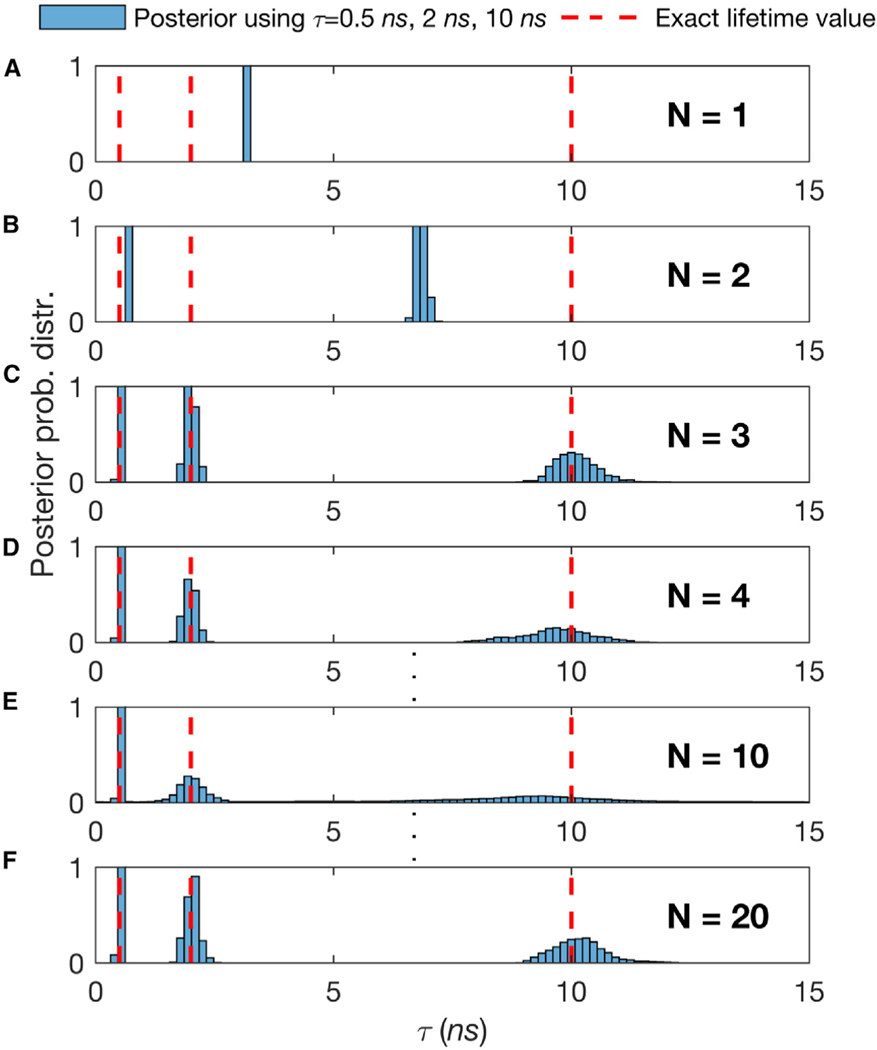
The Number of Species Assumed in the Analysis Directly Affects the Lifetimes Ascribed to Those Species, so an Independent Method Is Required to Estimate Species Numbers (A–F) We generate synthetic traces with 3 species with a total of 2 × 10^4^ photon arrivals and lifetimes, τ, of 0.5, 2, and 10 ns. To estimate the τ within the normal (i.e., parametric) Bayesian paradigm, we start by assuming the following number of species, *N=* 1 (A), *N=* 2 (B), *N=* 3 (C), *N=* 4 (D), … , *N=* 10 (E), … , and *N=* 20 (F). The good fit provided by *N* > 2 and the mismatch in the peak of the posterior distribution over the lifetime and correct value of the lifetime (red dotted line) in all others underscores why it will become critical for us, or any method analyzing single photon data in the context of confocal microscope experiments, to correctly estimate the number of species contributing to the trace to deduce chemical parameters such as lifetime.

We have previously exploited the Bayesian nonparametric (BNP) paradigm^75–78^ to analyze single-photon arrival time traces to learn diffusion coefficients from the minimal photon numbers drawn from single-spot confocal experiments.^10,79^ Traditionally, such photon arrivals were analyzed using tools from fluorescence correlation spectroscopy (FCS) in which very long traces were collected and auto-correlated in time. Just as with the problem at hand, the direct photon-by-photon analysis demanded a different approach, as the stochastic number of molecules contributing photons was unknown and an estimate of that number deeply affected our diffusion coefficient estimate. It is for this reason that we invoked the nonparametric paradigm there. In particular, the BNP paradigm is also preferred here on this basis: assuming an incorrect number of species, when these and their associated lifetimes are assumed unknown, leads to incorrect lifetime estimates for each species (see Figure 2). This further begs the question as to whether fits of the data with different, incorrect models can be compared in the first place.

Here, we propose a method that exploits BNPs^80^ to learn species and their associated lifetimes with as few photons as possible using pulsed illumination from a single illuminated spot. As with any inverse method, in BNPs we start from the data—namely the time lag between the peak of the pulse and the detection time of the photon, called “microtime,” discussed in more detail later in the article. To be precise, each species is defined as contributing photons sometime after pulsing dictated by an exponential distribution with a decay constant (lifetime) unique to that species. Just as we treat model parameters as random variables in the parametric Bayesian paradigm, within the BNP paradigm, we treat models themselves as the random variables and try to learn full posterior distributions over the number of species.

The advantages of using BNPs are 4-fold: (1) we can learn full posterior distributions over species present in the measurements, which is especially relevant for datasets with limited photons as the number of species becomes highly uncertain; (2) by resolving lifetimes and species from the raw photon arrivals directly in contrast to processed data that necessarily contains less information, we can minimize photodamage; (3) as a corollary to the previous point, we can monitor processes out of equilibrium in which only few photons are available before chemical conversion into another species; and (4) given long traces, we can exploit the additional data, if need be, to discriminate between species with small differences in lifetimes.

## RESULTS

### Aims of the Study

Our goal is to characterize quantities that describe molecular chemistry at the data-acquisition timescales of TCSPC, with a focus on obtaining lifetime estimates and the number of chemical species. To estimate lifetimes, we also estimate intermediate quantities, such as the fraction of different species contributing photons, as detailed in the Mathematical Formulation section.

Within the BNP approach,^81–83^ our estimates take the form of posterior probability distributions over unknown quantities. These distributions combine parameter values, probabilistic relations among different parameters, and the associated uncertainties. To quantify this uncertainty, we calculate a posterior variance and use this variance to construct error bars (i.e., credible intervals). As follows from Bayesian logic, the sharper the posterior, the more conclusive (and certain) the estimate.^79,81,84^

### Method Validation using Synthetic Data

**Figure 3.**
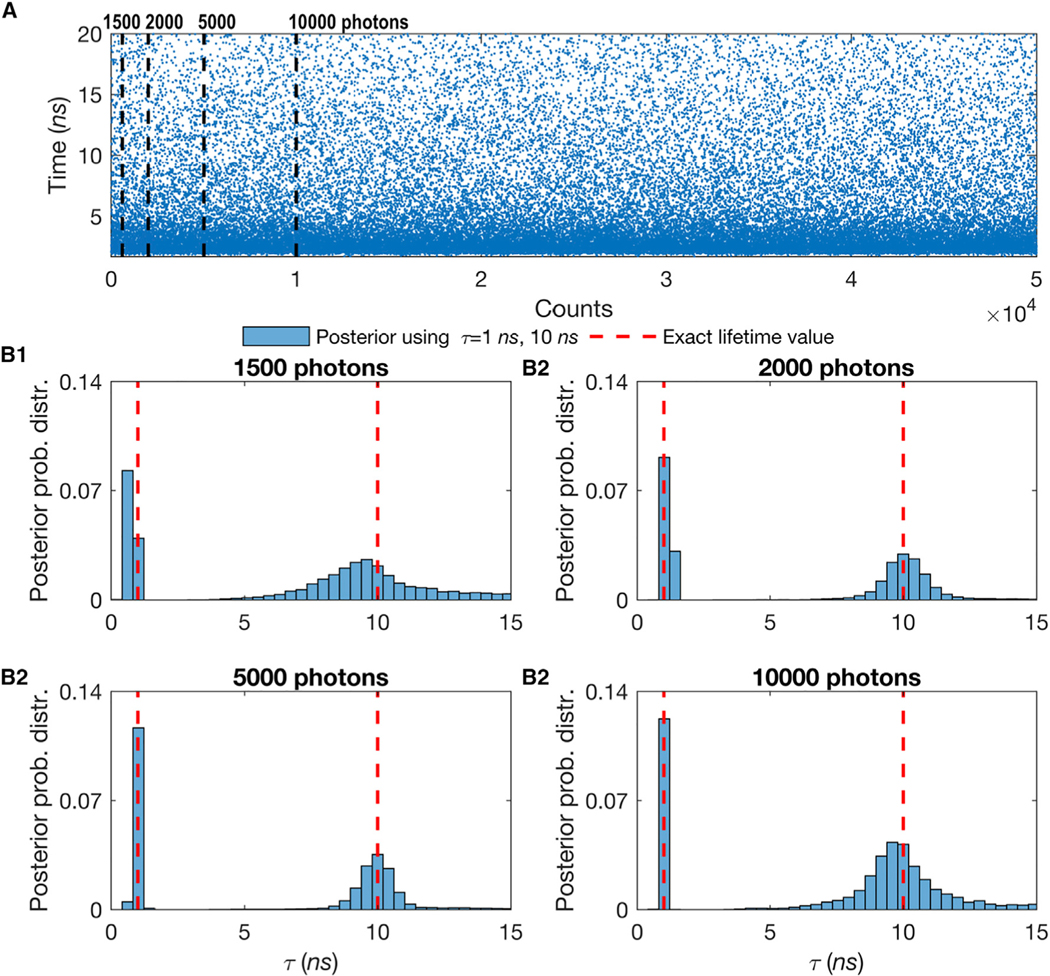
Effect of the Number of Detected Photons on 2 Simultaneous Molecular Lifetime Estimates Showing That the More Photons Collected, the Sharper the Lifetime Estimate for the Case of 2 Species (A) Here, we use mixtures of 2 species with different lifetimes, while all molecules are immobilized. The synthetic data are generated using τ = 1 ns for the first species and τ = 10 ns for the second with an equal ratio of molecules of each species (50%:50%). The blue dots represent single-photon arrival times detected after each excitation pulse. (B) In the analysis to determine both lifetimes, we start with just 1,500 photons (first dashed line in A) (B1) and gradually increase the number of photons to 2,000 (B2), 5,000 (B3), and 10,000 (B4) photons. Here, all of the other features such as the frequency of acquisition and width of pulse are the same as in Figure S1. Also, we follow the same dashed red line convention as in Figure S1. To see the results for more than two species see the Supplemental Experimental Procedures and Figures S5 and S6.

**Figure 4.**
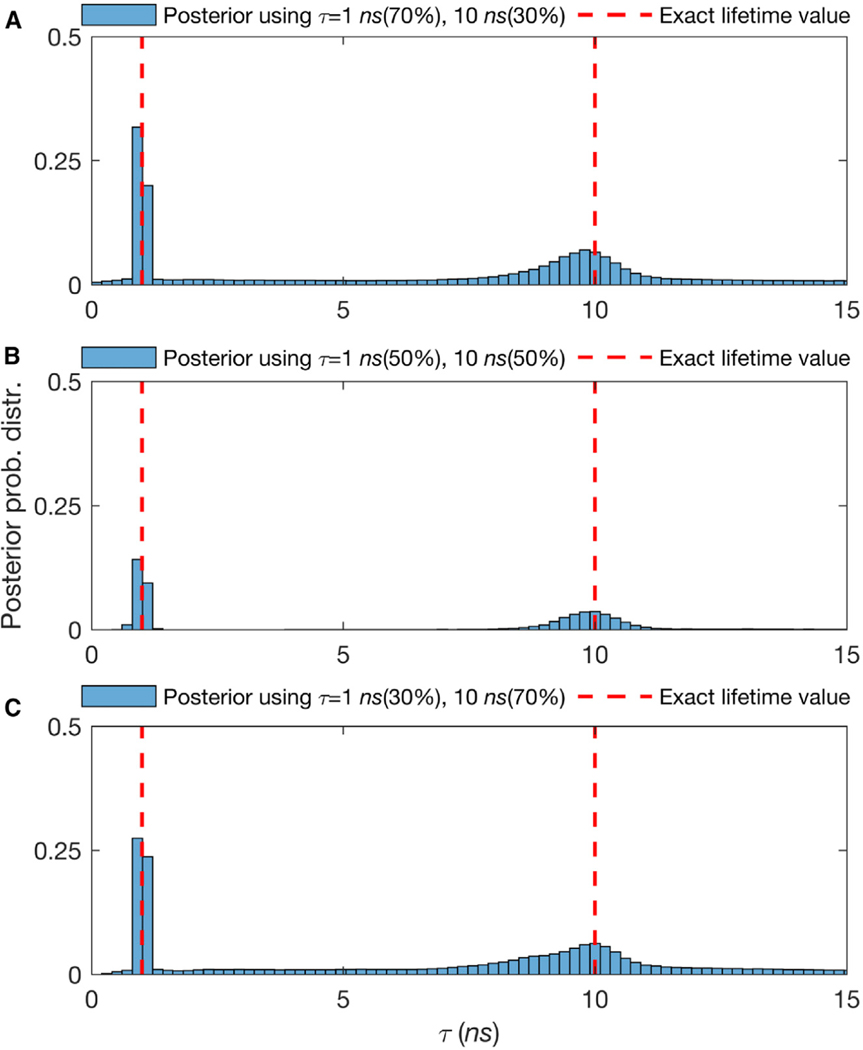
Effect of the Relative Fraction of Contributing Molecules from Different Species on Molecular Lifetime Estimates Showing That Higher Molecular Contributions Provide More Photons per Unit Time and Thus Sharper Lifetimes Estimates (A–C) The posterior probability distributions of traces with lifetimes of 1 and 10 ns, with 3,000 total photons and fraction of contributing molecules from different species of 70%–30%, 50%–50%, and 30%–70%, respectively. Here, all other features such as the frequency of acquisition and width of pulse are the same as in Figure S1. Also, we follow the same dashed red line convention as in Figure S1. For more details see the Supplemental Experimental Procedures and Figure S7.

**Figure 5.**
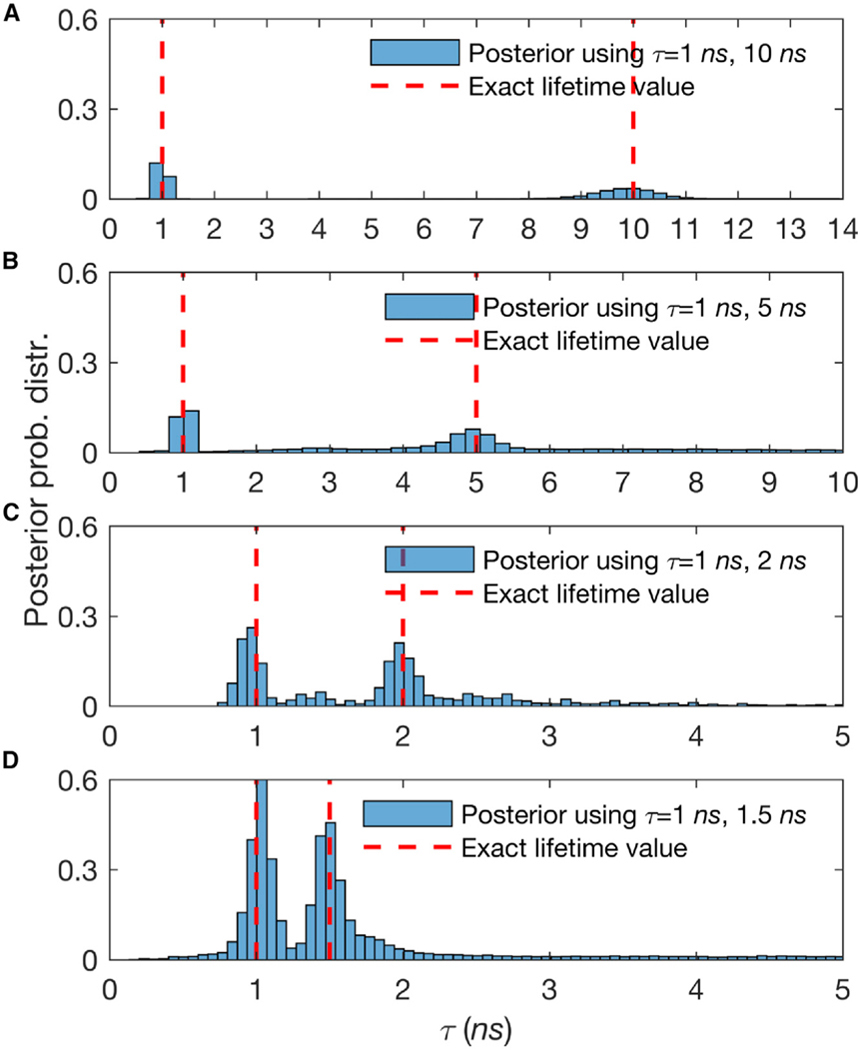
Lifetime Resolution for Double Species Lifetimes Posterior probability distributions over estimated lifetimes have been shown. The synthetic traces acquired contain 3,000–20,000 photon arrivals and start in (A) with well-separated lifetimes of 1 and 10 ns (≈ 3,000 photons) before gradually considering less-well-separated lifetimes such as in (B), where the lifetimes are 1 and 5 ns (≈ 3,000 photons), in (C), where the lifetimes are 1 and 2 ns (≈ 10,000 photons), and in (D), where the lifetimes are 1 ns and at last 1.5 ns (≈ 20,000 photons). The fraction of molecules contributing photons from different species is evenly split (50%−50%). Here, all of the other features, such as the frequency of acquisition and width of pulse, are the same as in Figure S1. Also, we follow the same dashed red line convention as in Figure S1.

To demonstrate the robustness of our method, we generate synthetic traces for immobilized molecules with (1) variable dataset sizes (Figure S1) involving multiple species (Figure 3); (2) a variable fraction of molecules contributing photons from different species (Figure 4); and (3) a variable difference of lifetimes for mixtures of lifetimes (Figure 5). All of the parameters not explicitly varied are held constant across all of the figures. The parameters not varied are held fixed at the following baseline values: lifetime between 1 and 10 ns, which is the typical lifetime range of a fluorophore^18,85^; 2 species, which is most frequent in related studies^18,19,23^; and fraction of molecules contributing photons from different species set at 50%:50%.

Also, as seen in the Supplemental Experimental Procedures, we worked with cases involving 3 and 4 different species (as opposed to just 1 or even 2 species), as this scenario presents the greatest analysis challenge because very few photons, and thus little information, are gathered on each species. In a similar spirit, we also defaulted to short traces that highlight the value of analyzing data in its rawest form. As the mathematics remain unaffected and this scenario reflects the reality of many experiments, we show in the Supplemental Experimental Procedures and Figures S2 and S3 the results for freely diffusive molecules.

### Number of Photons

We benchmark the robustness of our approach with respect to the length of the trace (i.e., the total number of photon arrivals) at a fixed number of species, lifetime, and molecule photon emission rate. For instance, to obtain an estimate of the lifetime within 10% of the correct result in the 1-species case, our method requires only ≈ 100 photons (emitted from the species of interest). In the case of 2 species, our proposed BNP approach requires only ≈ 3,000 photons (see Figures 3 and S1). To determine how many photons were required by our method, we chose the mean value of the lifetime posterior and measured the percentage difference of this mean to the ground truth known for these synthetic traces.

In general, the numbers of photons demanded by our method are minimal, although the absolute number depends on a broad range of experimental parameter settings. This is the reason why, throughout this work, we explore different settings—holding all other settings fixed—in subsequent subsections and the Supplemental Experimental Procedures.

Another important concept, illustrated in Figures 3 and S1, that will keep reappearing in subsequent sections is the concept of a photon as a unit of information. The more photons we have, the sharper our lifetime estimates. This is true, as we see in these figures, for increasing the trace length. Similarly, as we see in subsequent subsections, we also collect more photons as we increase the contribution of labeled molecules (and thus the number of molecules contributing photons to the trace).

### Mixtures of Different Species Contributing Photons

To test the robustness of our method when different species contribute an uneven number of photons, we simulated data with 70% of the population in species 1 and 30% in species 2 (Figure 4A). We also considered fractions of contributing molecules from different species of 50%:50% (Figure 4B), and 30%:70% (Figure 4C). For all of the cases, the lifetimes were fixed at 1 and 10 ns for ≈ 3,000 photon arrivals. Figure 4 summarizes our results and suggests that posteriors over lifetimes are broader—and thus the accuracy with which we can pinpoint the lifetimes drops—when the contribution of labeled molecules is lower. Intuitively, we expect this result, as fewer species within the confocal volume provide fewer photons, and each photon carries with it information that helps refine our estimated lifetimes. For more results, see the Supplemental Experimental Procedures and Figure S7.

### Lifetime Resolution

We repeat the simulations with 2 species, and ask how many photons are required to resolve similar lifetimes. Here, we present the dependency of the time resolution to the number of collected photons in Figure 5. As expected, the number of photons required to resolve increasingly similar lifetimes grows as the ratio of lifetimes approaches unity. However, this also suggests that if we were to resolve species of similar lifetimes, we could use the amount of data typically used in TCSPC or phasor analysis to resolve these, while TCSPC or phasor analysis would still require an additional order of magnitude more data. As we noted earlier, both TCSPS and phasor analysis must impose by hand the number of species, while, in our method, the number of species are learned. Moreover, if we know the number of species, we require even fewer photons than we mentioned earlier.

### Estimation of Physical Parameters from Experimental Data

To evaluate our approach on real data, we used experimental data collected under a broad range of conditions. We used measurements from different fluorophores, namely Cy3, TMR, Rhodamine-B (Rhod-B), and Rhod-6G. The lifetimes for these dyes are first benchmarked by fitting TCSPC photon arrival histograms from entire traces and compared them with published values.^86–89^

**Figure 6.**
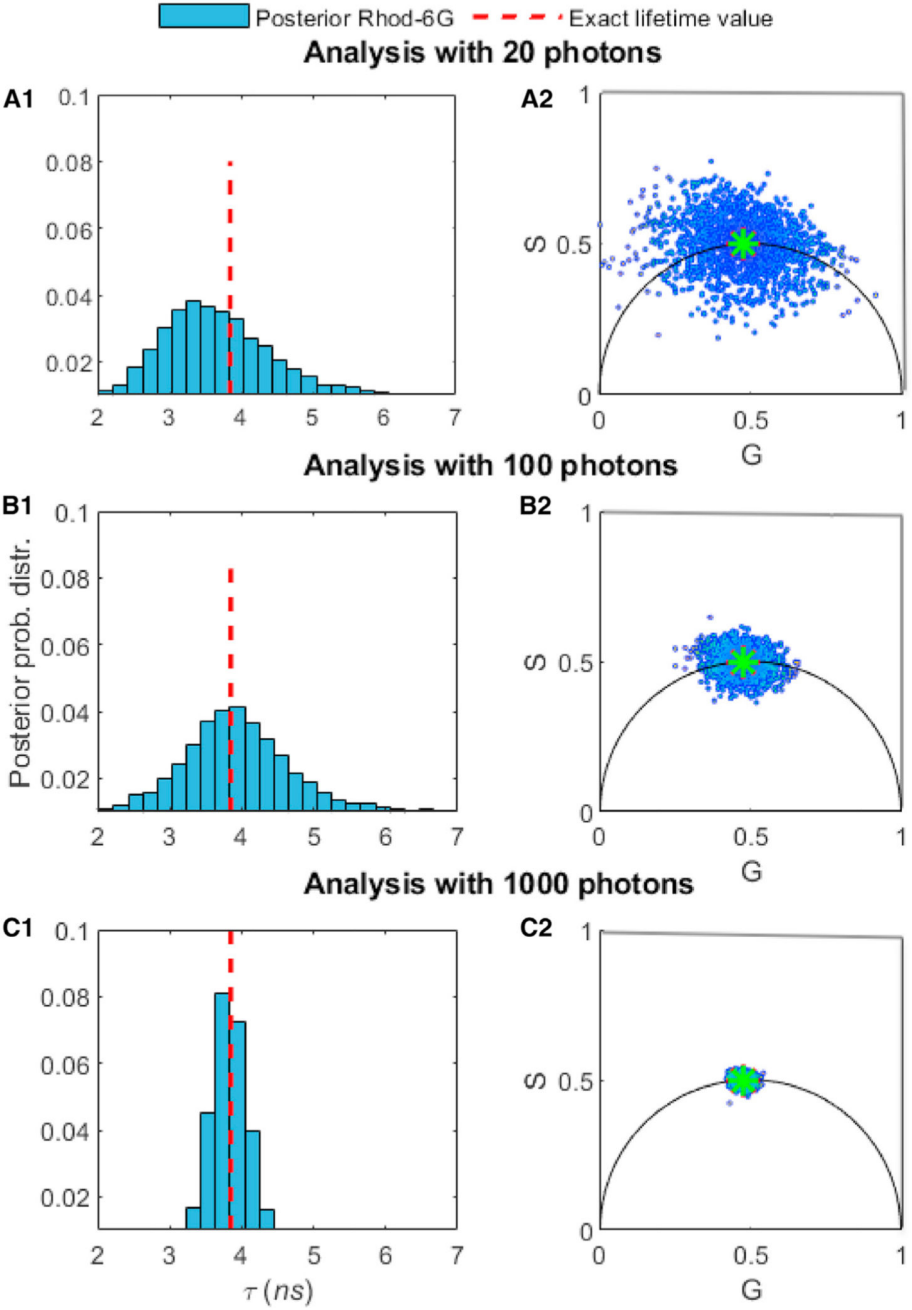
Comparison of the Number of Photons Needed to Assess the Lifetimes of Rhod-6G In (A1), we used 20 photons from experimental time trace Rhod-6G. For visualization purposes only, we show the corresponding phasor plots in (A2). In (B1) and (B2) and (C1) and (C2,) we repeated the analysis for 100 and then 1,000 photons. Using our method relying on BNPs, the estimated lifetimes are (A1) τ = 3.10 ns, (B1) τ = 3.95 ns, and (C1) τ = 3.91 ns. The excitation pulses occur at a frequency of 40 MHz and we assume a Gaussian shape with a standard deviation of 0.1 ns. The ground truth (dashed red lines) is obtained using TCSPC photon arrival histogram fitting when analyzing the whole time trace. In our BNP analysis, we do not pre-specify the number of species; we learn them alongside the associated lifetimes.

**Figure 7.**
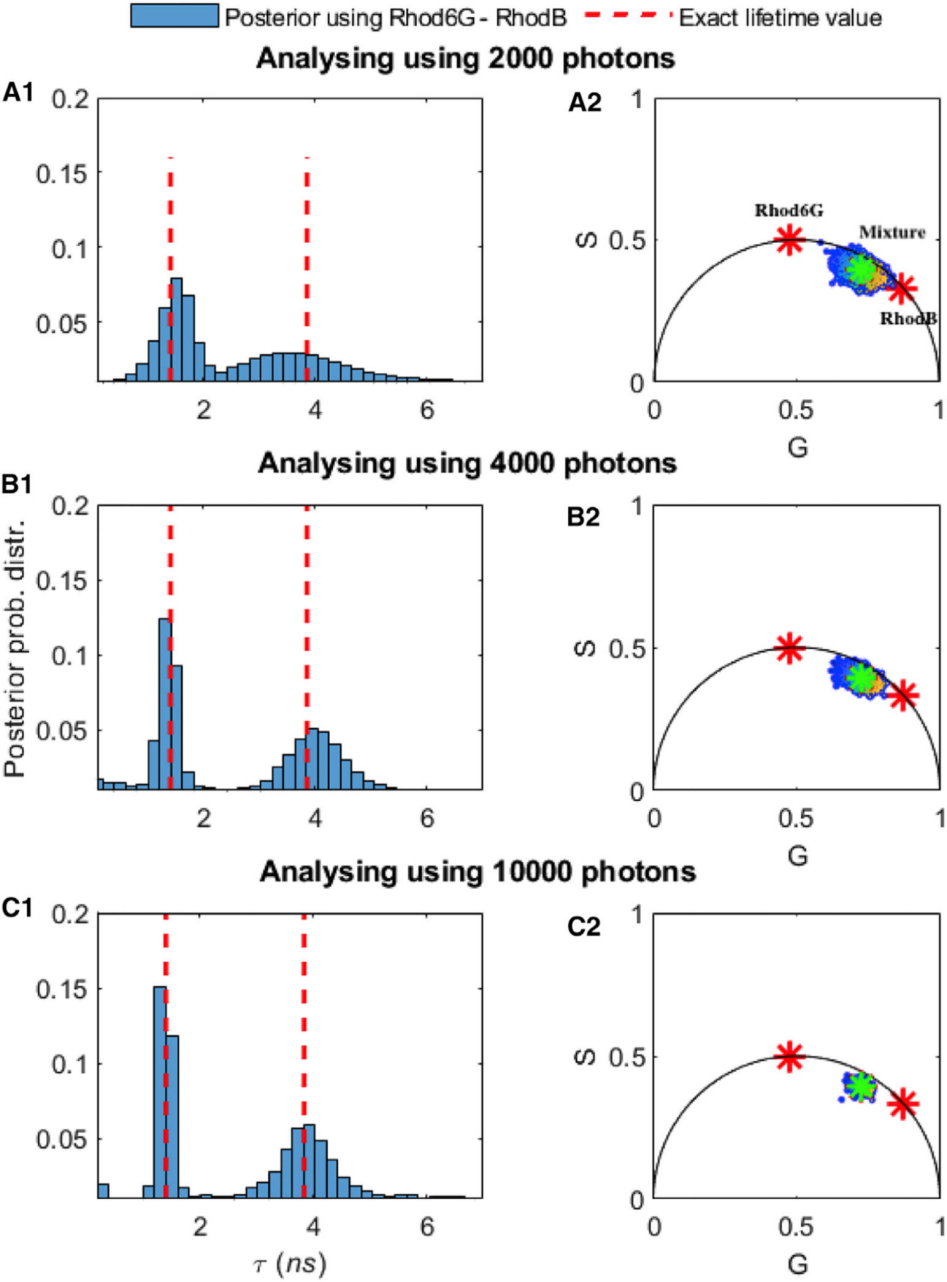
Comparison of the Number of Photons Needed to Assess the Lifetimes of Mixtures of Rhod-B and Rhod-6G In (A1) we used 2,000 photons. For visualization purposes only, we show the corresponding phasor plots in (B1). In (B1) and (B2) and (C1) and (C2,) we repeated the analysis for 4,000 and then 10^4^ photons. Using BNPs, the estimated lifetimes are (A1) τ = 1.44–3.39 ns, (B1) τ = 1.42–3.96 ns, and (C1) τ = 1.41–3.90 ns. Here, all of the other features such as the ground truth (dashed red lines), frequency of acquisition, and so forth are the same as in Figure 6. The green star in (A2)–(C2) is the location of mixture of 2 species when we use whole trace, and the red asterisks show the location of the single species lifetime, for visualization purposes only, whose lifetimes we independently know from experiments on individual species.

**Figure 8.**
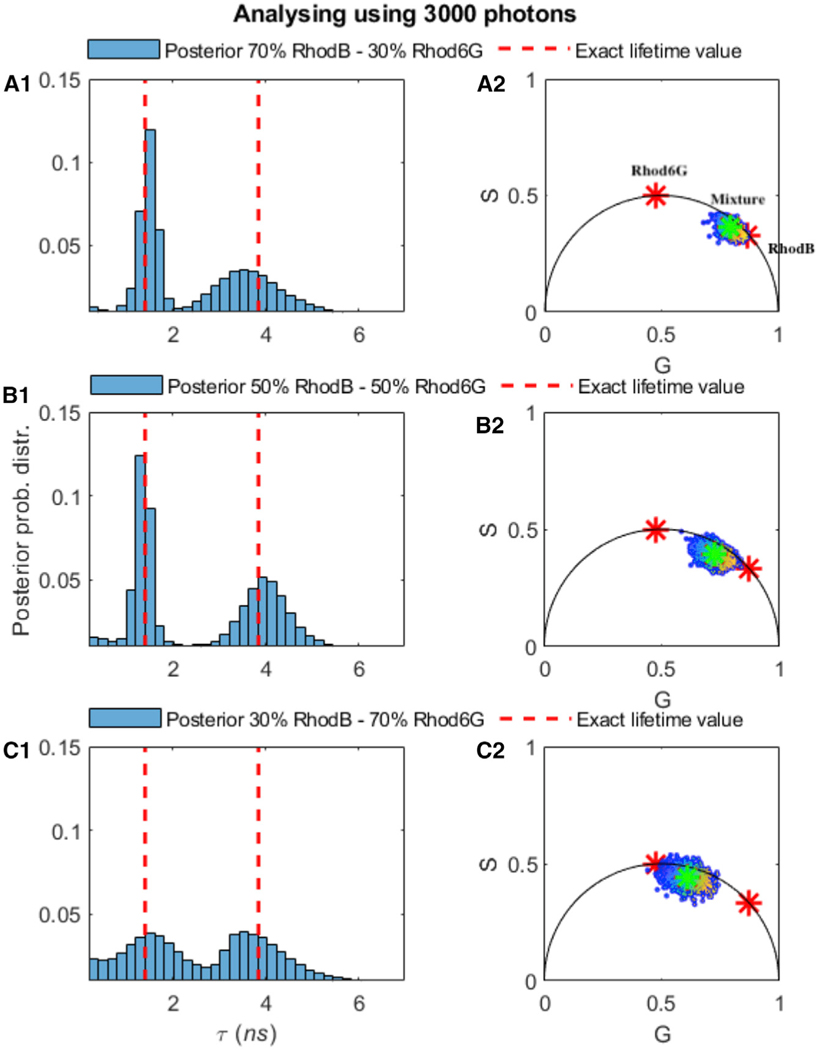
Effect of the Fraction of Molecules Contributing Photons from Different Species on Molecular Lifetime Estimates Showing That Higher Molecular Contributions Provide More Photons per Unit Time and Thus Sharper Lifetime Estimates Higher molecular contributions provide more photons per unit time and thus sharper lifetime estimates. The experimental trace is selected using 2 species, Rhod-B and Rhod-6G, with a total of ≈ 3,000 photon arrivals, with a different fraction of photons derived from different species (70%–30%) (A1), 50%–50% (B1), and 30%–70% (C1). The estimated lifetimes using BNPs are (A1) τ = 1.44–3.39 ns, (B1) τ = 1.42–3.96 ns, and (C1) τ = 1.41–3.90 ns. Here, all of the other features such as the ground truth (dashed red lines), frequency of acquisition, and so forth are the same as in Figure 6. The green and red asterisks on subfigures (A2)–(C2) are explained in the Figure 7 caption.

Figures 6, 7, and 8 were collected using the Rhod-B and Rhod-6G dyes, and these results were used to benchmark the robustness of our method on individual species and mixtures of species with a variable fraction of chemical species contributing photons. In Figure S8, we show more experimental results for cases involving >2 species.

In Figure 6, we verified our method on Rhod-6G with respect to the total number of photon arrivals. The first important conclusion is that we need ≈ 100 photons to obtain an estimate of the lifetime within 10% of the correct result (as obtained from our benchmark). For ≥2 species, the situation for phasor analysis, TCSPC photon arrival histogram fitting, or direct analysis of photon arrivals using parametric Bayesian methods or maximum likelihood grows more challenging. The number of species cannot be independently determined, and assuming an incorrect number of species leads to incorrect lifetime estimates (see Figure 1 for phasors and Figure 2). Moreover, for all of the cases, we could reliably determine the ground truth (dashed red lines in Figure 2) from the TCSPC photon arrival histogram fitting when using the whole trace with all of the photons available. To be clear, we learn the number of species directly using BNPs and do not assume a number ahead of time.

Again, the absolute number of photons demanded by our method depends on a broad range of experimental parameter settings. This is the reason why we explore different settings—holding all other settings fixed—just as we did with synthetic data in subsequent subsections and the Supplemental Experimental Procedures.

### Benchmarking on Experimental Data using a Different Number of Photons for Mixtures of Rhod-B and Rhod-6G

Similar to the synthetic data analysis appearing in Figure 3, we benchmarked the robustness of our approach with respect to the length of the trace (i.e., the total number of photon arrivals), given fixed lifetimes and fraction of chemical species contributing equal numbers of photons (50%:50%). The important message here is that, for the values of parameters selected, we need ≈ 100 photons for single species (Figure 6) and ≈ 3,000 photons for double species (Figures 7 and 8). For instance, to obtain an estimate of the lifetime to within 10% of the correct result for the case of 2 species, our method requires ≈ 3,000 photons.

### Benchmarking on Experimental Data using Different Fractions of Rhod-B and Rhod-6G

We start by evaluating our method on mixtures of Rhod-B and Rhod-6G, but present in different amounts. Similar to Figure 4 for the analysis of 2 species from synthetic data, we show estimates of the lifetimes for 2 species, Rhod-B and Rhod-6G, present at a 70%:30% fraction (Figure 8A), at a 50%:50% fraction (Figure 8B), and at a 30%:70% fraction (Figure 8C). Figure 8 (and Figure S9) summarize our results and suggest that posteriors over lifetimes are broader—and thus the accuracy with which we can pinpoint the lifetimes drops—when the contribution from the dye concentration for that species is lower. To obtain an estimate of the lifetime to within 10% of the correct result, our method requires ≈ 3,000 photons directly emitted from the dye; for visualization purposes, the corresponding phasor plot is provided in Figure 8. In the Supplemental Experimental Procedures, we show additional results for the case of 3 and 4 species, which are additionally challenging for existing methods with different fractions of chemical species contributing photons.

## DISCUSSION

Across all spectroscopic and imaging applications, the photon is the basic unit of information.^79,90^ Decoding information directly from single-photon arrivals, with as few photons as possible without binning or correlating or other pre-processing of the data, is the main focus of our data-centric analysis strategy. However, decoding information directly from single-photon arrivals presents fundamental model selection problems.

For example, in the case of FCS, if we are to learn diffusion coefficients directly from limited photon arrivals, we must know how to write down a likelihood; put differently, we must know the number of molecules contributing photons that, in turn, dictate the form for the likelihood.^79^ As we do not know how many molecules we have and what the appropriate likelihood should be, we have a model selection problem. Similarly, for lifetime imaging, if we are to learn the lifetime of the chemical species contributing photons, then we must also know the number of species to write down a conventional likelihood.

Traditional Bayesian methods do not have a direct solution to the model selection problem,^80,82^ as they also require us to be able to write down a likelihood. That is, they consider a fixed model (and a fixed likelihood) and treat the model’s parameters as random variables of the posterior distribution. By contrast, BNPs, which are a direct logical extension of parametric Bayesian methods, treat models alongside their parameters as random variables.^75,83,91–96^

This ability to treat models themselves as random variables is the key technical innovation that prompted the development of BNPs in the first place. BNPs make it possible to avoid the computationally infeasible task of enumerating and then comparing all of the models for any associated parameter values to all other competing models and their associated parameter values.

The BNP approach to tackling lifetime image analysis that we propose here cannot replace phasor analysis^20,23,60,62,64,69,97^ or TCSPC photon arrival analysis under an assumed number of species^2,14,29,38,40,98^ for simple 1-component systems on account of their computational efficiency. However, at an acceptable computational cost, BNP approaches provide a powerful alternative. They give us the ability to determine the number of species (and probabilities over them if the data are uncertain due to their sparsity or otherwise); use much less data to obtain lifetime estimates (and thus reduce phototoxic damage to a light-sensitive sample); use longer photon arrival time traces, if available, to tease out small differences in lifetimes between species, as BNP-based methods are more data efficient; probe processes resolved on faster timescales (again, as we require minimal photon numbers); and exploit all of the information encoded in the photon arrivals (and thus not require separate control experiments, as often needed in phasor approaches, for the measurement of the lifetime of one species to determine the lifetime of a second species when a mixture of 2 species, for example, is present).

As for the computational cost, obtaining lifetimes (to within 10% of the ground truth lifetime for 1 species for the parameters we used in Figures S1 and S6 requiring ≈ 100 photons) takes 5 min on a typical scientific desktop as of the publication date of this article (based on a system with 6G RAM, Core (TM) i7–2.67 GHz CPU). For a 2-species mixture, Figures 3 and 7, under the same parameters and requiring 3,000 photons, it was a modest increase to 15 min. The point here is that the analysis of single or multispecies data can be performed with an average desktop computer, and it does not necessarily require high-performance computing facilities.

The real strength of BNP becomes clear when we reach 2, 3, 4, or possibly even more species. Beyond being able to work with low photon counts, another key advantage of our method is its flexibility. The ability to use BNP and treat models as random variables in lifetime imaging is the real point here, and, as such, our framework can be adapted to treat a range of experimental setups.

In particular, our framework can straightforwardly be adapted to treat any instrumental response function (IRF) by modifying Equation 4, as appropriate, and any background photon arrival statistics or detector dark counts by modifying Equation 5 especially as relevant to *in vivo* imaging. In Figure S4, we tested the robustness of our method by varying the number of background photons in our dataset. More significant extensions of our work, albeit generalizations that would leverage the framework at hand, would be to consider lifetime changes, due to chemical modifications of our species, over the timescale of data acquisition, as may be expected in complex *in vivo* environments.^99,100^ Another is to extend our work to analyze fluorescence lifetimes over multiple spatial locations, the purview of fluorescence lifetime imaging (FLIM) analysis.^72,101–104^ Finally, we could also generalize our proposed method to accommodate non-exponential lifetime decays if such decay probabilities are warranted by the data by modifying Equation 5.

These and further generalizations that can be implemented within a BNP framework highlight the flexibility afforded by BNPs and the nature of what can be teased out from challenging datasets. BNPs themselves suggest productive paths forward to tentatively formulate inverse strategies for challenging datasets not otherwise amenable to traditional, parametric Bayesian analysis.^105^

## EXPERIMENTAL PROCEDURES

### Materials Availability

#### Lead Contact

The Lead Contact is Steve Pressé (spresse@asu.edu).

#### Materials Availability

This study did not generate new unique reagents.

#### Data and Code Availability

All code is available on the Lead Contact’s website (https://cbp.asu.edu/content/steve-presse-lab) and upon request (Massachusetts Institute of Technology [MIT] license). Data can be made available upon request by contacting the Lead Contact.

### Mathematical Formulation

Here, we describe the mathematical formulation of our analysis method of time-resolved pulsed excitation single-photon arrival data. For clarity, we focus on measurements obtained on a fluorescence setup that use a train of identical excitation pulses. Following each pulse, ≥1 molecules located near the illuminated region may be excited from their ground state. As the excited molecules decay back to their ground state, they may emit photons and we record the detection time. Below, we describe how we analyze such recorded times.

We start from single-photon detection times, which consist of the raw output in a time-resolved pulsed excitation single-photon arrival experiment. Similarly, these are measured based on the time difference between excitation pulses, which are time stamped, and the detection time of the first photon arriving after each pulse.^18,39,106^ Precisely, our raw input is *Δt* = (*Δt*_1_, *Δt*_2_, …, *Δt*_*k*_), where *Δt*_*k*_ is the time interval between the preceding pulse’s time and the photon detection time of the *k*^*th*^ detection. In the literature, each *Δt*_*k*_ is often called microtime. As some pulses may not lead to a photon detection, in general the microtimes in *Δt* are fewer than the total number of pulses applied during an experiment.

### Model Description

We assume that, once excited, each molecule remains excited for a time period that is considerably lower (typically a few nanoseconds) as compared to the time between 2 successive pulses (typically >4 times of the longest decay time in the sample^18^). This condition allows us to consider that any photon that is detected stems from an excitation caused by the very previous pulse and not from earlier pulses. Also, as excitation pulses in time-resolved pulsed excitation single-photon arrival experiments are weak,^38,98^ and typically 1 in ≈ 100 pulses results in a photon detection,^18^ we ignore, to a very good approximation, multiple photon arrivals. As the number of detected photons coming from the background is considerably lower than the number of detected photons coming from the excited molecules, typically 1 to ≈ 1,000, we also ignore background photons. However, background photons can be dealt with straightforwardly by modifying Equation 7 to incorporate the effect of background in the model.

To analyze the recordings in *Δt*, we assume that the sample contains in total *M* different molecular species that are characterized by different lifetimes τ_1_, …, τ_*M*_. Since molecules of each species may be excited by the pulses with different probabilities (because of different fraction of molecules contributing photons from different species), we consider a probability vector $\bar{\pi }=\left(\pi_{1},\ldots ,\pi_{M}\right)$ that gathers the probabilities of each species, giving rise to a photon detection. Allowing *s*_*k*_ to be a tag attaining integer values 1, …, *M*, that indicates which species triggered the *k*^*th*^ detection, we may write

(Equation 3) $$s_{k}|\bar{\pi }∼{\;\text{Categorical}\;}_{1:M}(\bar{\pi }).$$

The above equation reads as follows: “the tag *s*_*k*_ given $\bar{\pi }$ is a random variable sampled from a categorical distribution.” The categorical distribution is the generalization of the Bernoulli distribution, which allows for >2 outcomes.^107–109^ With this convention, the lifetime of the molecule triggering the *k*^*th*^ detection is τ_*sk*_. Of course, the number of molecular species *M* and the precise values of the lifetimes τ_1_, …, τ_*M*_ are unknown, and our main task is to estimate them using the recordings in *Δt*.

**Figure 9.**
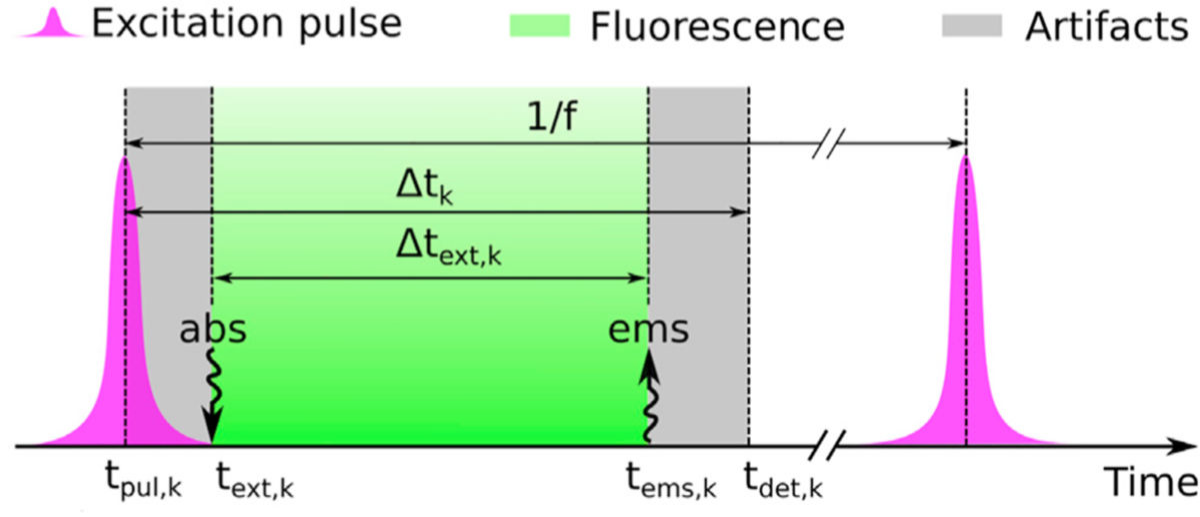
Factors That Contribute to the Recorded Photon Arrival Times Here, *t*_*pul.k*_ is the time of the pulse’s peak. Since pulses last for some time, they may excite the molecules at slightly different times. As such, we denote with *t*_*ext.k*_ the absorption time of the molecule triggering the *k*^*th*^ detection. Moreover, we denote with *t*_*ems.k*_ the emission time of the photon triggering the *k*^*th*^ detection. At last, on account of electronics limitations, the detection time, which we denote with *t*_*det.k*_, may be different from *t*_*ems.k*_. Here, the artifacts shown in gray originate from 2 sources: the left gray-shaded region is due to the width of the pulse, which leads to variation in the time of the molecular excitation, and the right gray-shaded region arises from the camera-dependent detection uncertainty. The time during which the fluorophore is excited (fluorescence lifetime) is shown in green. For more details, see also the Supplemental Experimental Procedures and Figure S12.

For clarity, we denote with *t*_*pul,k*_ the application time of the pulse that triggers the *k*^*th*^ photon detection. More precisely, *t*_*pul,k*_ is the time of the pulse’s peak. Because, in general, pulses last for some non-zero duration, and thus they may excite the molecules at slightly different times, we denote with *t*_*ext,k*_ the absorption time of the molecule triggering the *k*^*th*^ detection. Furthermore, we denote with *t*_*ems,k*_ the emission time of the photon triggering the *k*^*th*^ detection. Finally, due to the measuring electronics, the detection time, which we denote with *t*_*det,k*_, may be different from *t*_*ems,k*_; see Figure 9 for more details.

With this convention, our measured output consists of the time lags *Δt*_*k*_ = *t*_*det,k*_ — *t*_*pul,k*_. These time lags include (1) the time until absorption occurs, *t*_*ext,k*_ — *t*_*pul,k*_; (2) the time until fluorescence emission occurs, *t*_*ems,k*_ — *t*_*ext,k*_; and (3) delays and errors introduced by the measuring electronic devices, *t*_*det,k*_ — *t*_*ems,k*_. Below, we denote the middle period with *Δt*_*ext,k*_ = *t*_*ems,k*_ — *t*_*ext,k*_, while we denote with *Δt*_*err,k*_ = (*t*_*ext,k*_ — *t*_*pul,k*_) + (*t*_*det,k*_ — *t*_*ems,k*_) the sum of the others. From these 2, *Δt*_*ext,k*_ is the time the molecule spends in the excited state, while *Δt*_*err,k*_ gathers any artifacts caused by our setup either in the excitation or the detection pathway. The advantages of considering these 2 periods separately, as we explain below, is that (1) these represent independent physical processes and (2) each one is theoretically and experimentally characterized well.^18^

In particular, *Δt*_*err,k*_ is characterized by the IRF that, in each setup, is readily obtained with calibration measurements.^18^ In this study, we approximate the IRF as a Gaussian (Figure S11)

(Equation 4) $$\text{Δ}t_{err\;,k}∼\;\text{Normal}\;\left(\tau_{IRF},\sigma_{\text{IRF}\;}^{2}\right).$$

In this approximation, τ_IRF_ is the IRF’s peak time and σ_IRF_ = FWHM/2.355, where FWHM is the IRF’s full width at half-maximum. In the Supplemental Experimental Procedures, we explain the IRF’s calibration in detail.

Upon excitation, the time the molecule remains excited, *Δt*_*ext,k*_, is memoryless,^18^ and so follows the exponential distribution. Therefore,

(Equation 5) $$\text{Δ}t_{ext\;,k}|\lambda_{s_{k}}∼\;\text{Exponential}\;\left(\lambda_{s_{k}}\right)$$

where $\lambda_{s_{k}}$ is the inverse lifetime of the molecule triggering the detection of $\text{Δ}t_{\text{ext,}\;k}.$ Of course, the inverse lifetime depends upon the lifetime by $\lambda_{s_{k}}=1/\tau_{s_{k}}$.

Because *Δt*_*ext,k*_ and *Δt*_*err,k*_ are independent variables, the statistics of our measurements, which are given by $\text{Δ}t_{k}=\text{Δ}t_{ext,k}+\text{Δ}t_{err,k}$, follow

(Equation 6) $$\text{Δ}t_{k}|\lambda_{s_{k}}∼\text{Normal}\left(\tau_{\text{IRF}},\sigma_{\text{IRF}}^{2}\right)*\text{Exponential}\left(\lambda_{s_{k}}\right)$$

where * denotes a convolution^110^ and specifically has the probability density

(Equation 7) $$p\left(\text{Δ}t_{k}|\lambda_{s_{k}}\right)=\frac{\lambda_{s_{k}}}{2}\exp\left[\frac{\lambda_{s_{k}}}{2}\left(2\left(\tau_{\text{IRF}}-\text{Δ}t_{k}\right)+\lambda_{s_{k}}\sigma_{\text{IRF}}^{2}\right)\right]\text{erfc}\left(\frac{\tau_{\text{IRF}}-\text{Δ}t_{k}+\lambda_{s_{k}}\sigma_{\text{IRF}}^{2}}{\sigma_{\text{IRF}}\sqrt{2}}\right)$$

where erfc(•) denotes the complementary error function. In the Supplemental Experimental Procedures, we show analytically how Equation 7 arises from Equations 4 and 5.

In the next section, we describe how Equations 3 and 7 can be used in conjunction with BNP to obtain the estimates we are after.

### Model Inference

**Figure 10.**
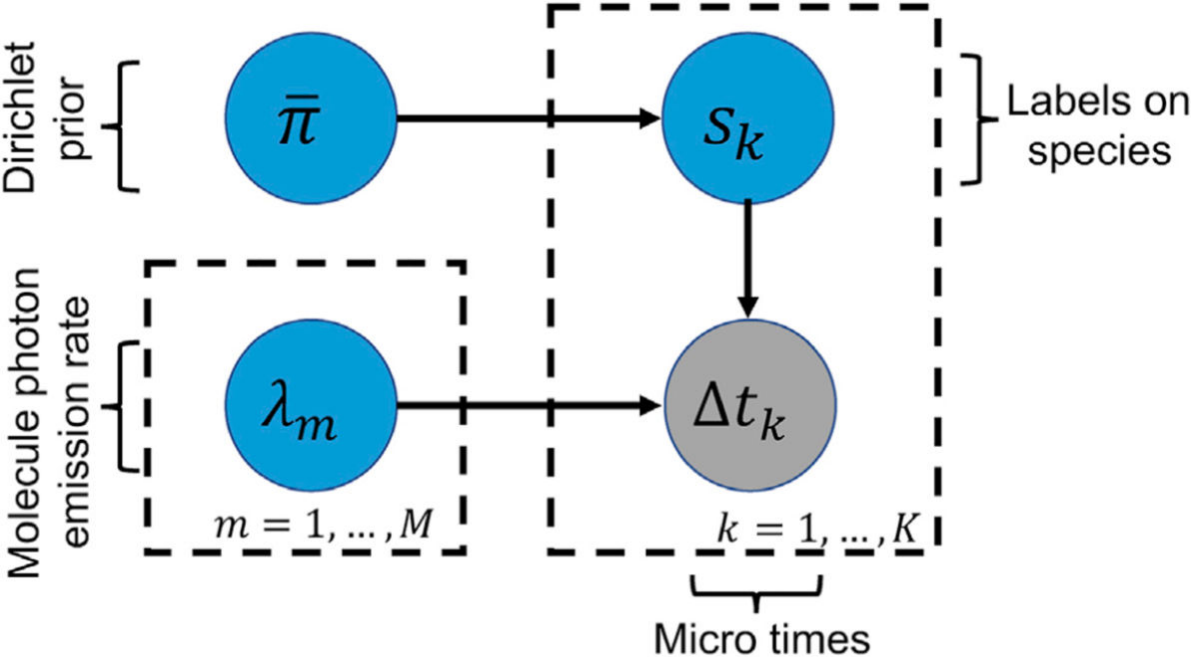
Graphical Representation of the Proposed Model A simple graphical representation of the model, where *Δt*_*k*_ is the microtime *k* with *k* = 1, …, *K*. The inverse lifetime of species *m* is shown by λ_*m*_, *m* = 1, …, *M*. The label *s*_*k*_ tells us which of the species is contributing the *k*^*th*^ photon. In the graphical model, the measured data are denoted by gray-shaded circles and the model variables, which require priors, are designated by blue circles. Each one of the labels has a prior, which is a Dirichlet probability $\bar{\pi }$.

All of the quantities that we wish to infer, for example, the species inverse lifetimes λ_1_, …, λ_*M*_ and excitation probabilities in $\bar{\pi }$, are represented by model variables in the preceding formulation. We infer values for these variables within the Bayesian paradigm.^80,82,84^ Accordingly, on the inverse lifetimes we place independent priors

(Equation 8) $$\lambda_{m}∼\text{Gamma}\left(\alpha_{\lambda },\beta_{\lambda }\right),\;m=1,\ldots ,M$$

that ensure strictly positive values. Here, for convenience only, we consider priors on inverse lifetimes where $\tau_{m}=\left(1/\lambda_{m}\right)$ is the molecular lifetime and λ_*m*_ is the inverse lifetime of species *m*. As the total number of species contributing photon detections in an experiment is unknown, we consider a symmetric Dirichlet prior^80,83^ (which is conjugate to the Categorical) on $\bar{\pi }$ of the form

(Equation 9) $$\bar{\pi }∼\text{Dirichle}t_{M}\left(\frac{\alpha }{M},\ldots ,\frac{\alpha }{M}\right)$$

where α is a positive scalar hyper-parameter. A graphical summary of the whole formulation is shown in Figure 10.

The distribution in Equation 9 ensures that $\bar{\pi }$ are valid probability vectors. Furthermore, Equation 9 is specifically chosen to allow for a large, M→∞, number of species. This is particularly important because the total number of molecular species contributing to the detections in TCSPC or FLIM experiments are typically unknown, and thus choosing a finite *M* may lead to underfitting. Specifically, at the limiting case M→∞, the prior on Equation 9, combined with Equation 3, results in a Dirichlet process.^75,83,111,112^ In other words, provided that *M* is sufficiently large, the estimates obtained through our model are independent of the particular value chosen (i.e., overfitting cannot occur).

With the nonparametric model just presented, although the total number of model molecular species is infinite, the actual number of molecular species contributing photons to the measurements is finite. Specifically, the number of contributing species coincides with the number of different tags *s*_*k*_ associated with *Δt*. In other words, instead of asking how many species contribute to the measurements, with our model, we ask how many of the represented species actually contribute at least one photon. Furthermore, instead of asking what the lifetimes are of these species, we ask what the lifetimes are of the species contributing at least one photon. Of course, as we estimate inverse lifetimes instead of lifetimes, we obtain the latter by $\tau_{m}=1/\lambda_{m}$.

With these priors, we form $p\left(\bar{\pi },s_{1},\ldots ,s_{K},\lambda_{1},\lambda_{2},\ldots |\Delta t\right)$, which is the joint posterior probability distribution that includes all unknown variables. To compute this posterior, we develop a Markov chain Monte Carlo (MCMC) scheme^84,113^ that generates pseudo-random samples with the appropriate statistics. The scheme is described in the Supplemental Experimental Procedures and a working implementation is also provided.

### Acquisition of Synthetic Data

The synthetic data presented in this study are obtained by standard pseudo-random computer simulations^114–118^ that simulate a common fluorescence lifetime imaging modality with a conventional single-spot confocal setup. Furthermore, in the simulations, we consider confocal regions created with pulsed excitation. To generate data mimicking as closely as possible the measurements obtained in real experiments, we simulate freely diffusing molecules of different species characterized by different diffusion coefficients and lifetimes. Details and parameter choices are provided in Tables S1, S2, and S3.

### Acquisition of Experiment Data

The synthetic data presented in this study are obtained as described below.

### Sample Preparation

Sample solutions of Rhodamine B (Rhod-B, Wako Pure Chemical Industries), Rhodamine 6G (Rhod-6G, Sigma-Aldrich), tetramethylrhodamine-5-maleimide (TMR, Invitrogen), and Cy3 monofunctional NHS-ester (Cy3, GE Healthcare) were prepared with Milli-Q water at a 1-μM concentration. Nonionic surfactant (0.01% Triton X-100) and 2 mM Trolox were added to prevent the adsorption of dye molecules to the glass surface and reduce photophysical artifacts, respectively.

### Experiments

Fluorescence lifetime measurements were carried out using a confocal fluorescence microscope with a super continuum laser (Fianium SC-400–4, frequency of 40 MHz). The output of the laser was filtered by a bandpass filter (Chroma Technology D525/30 m) and focused onto the sample solution using a 60 × objective lens (Nikon Plan Apo IR) with a numerical aperture (NA) of 1.27. The excitation power was set at 0.3 μW at the entrance port of the microscope. Fluorescence photons were collected by the same objective lens and guided through a confocal pinhole as well as a bandpass filter (Chroma Technology D585/40 m), and then detected by a hybrid detector (Becker & Hickl HPM-100–40-C). For each photon signal detected, the routing information was appended by a router (Becker & Hickl HRT-82). The arrival time of the photon was measured by a TCSPC module (Becker & Hickl SPC-140) with the time-tagging mode.^37^ The time resolution was evaluated by detecting the scattering of the incident laser light at a cover glass, and it was typically 180 ps at FWHM.

## Supplementary Material

SI2

## HIGHLIGHTS

Bayesian nonparametrics approach proposed for estimation of the lifetime

Using this approach, the number of fluorescence species is also learned

Use of direct photon-by-photon analysis of data drawn from pulsed excitation experiments

Method benchmarked on simulated and experimental data for up to 4 species
