# Supplementary material for: Direct Photon-by-Photon Analysis of Time-Resolved Pulsed Excitation Data using Bayesian Nonparametrics
Source: Cell Rep Phys Sci. Author manuscript; Available in PMC 2021 Aug 18. (PMC8373049; doi:10.1016/j.xcrp.2020.100234)
Supplement: SI2 [file NIHMS1724252-supplement-SI2.pdf]

Cell Reports Physical Science, Volume *1*

Supplemental Information

Direct Photon-by-Photon Analysis of Time-Resolved Pulsed Excitation Data using Bayesian Nonparametrics

Meysam Tavakoli, Sina Jazani, Ioannis Sgouralis, Wooseok Heo, Kunihiko Ishii, Tahei Tahara, and Steve Pressé

Supplemental Experimental Procedures

In this supplement, we present additional analyses and technical details expanding upon the material presented in the main text. These include: (i) additional analysis of synthetic and experimental traces that include the estimation of lifetimes and the fraction of different species contributing photons; (ii) additional details on the theoretical approaches used; and (iii) a complete description of the inference framework developed that includes choices for prior probability distributions and a computational implementation. Moreover, in our BNP analysis, we do not pre-specify the number of species, we learn them.

Additional results

Analysis of additional synthetic data

In the main text we focused on the estimation of: lifetime, τ, with values less than 10 ns which are typical lifetime values in *in vivo* applications [1]. Here, we explore broader parameter ranges from freely diffusive molecules, Figs. S2 and S3 to the case when we have different background photons, Fig. S4, which we evaluated our method respect to different background levels to see how it behaves with different number background photons. Moreover, we evaluated our method for cases with more than two species, Figs. S5 and S6, and estimate the fraction of molecules contributing photons from different species, Fig. S7, that we explain in the main text in Section "Mixtures of different species contributing photons" .

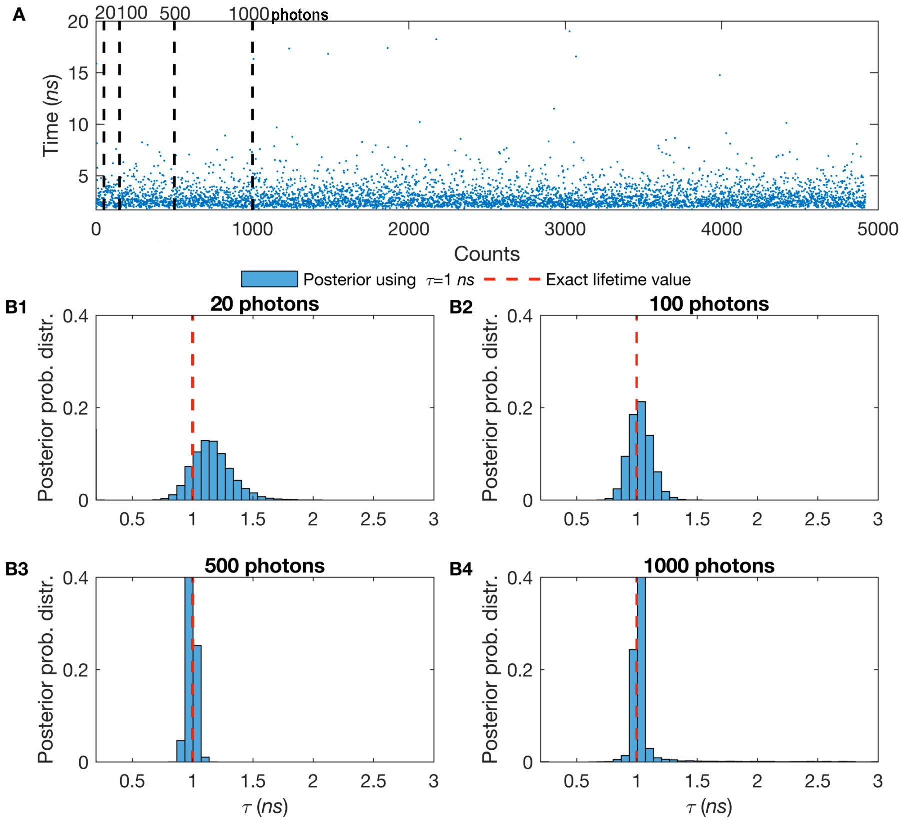

Figure S1: **The greater the number of detected photons, the sharper the molecular lifetime estimate.** (A) Here, we work on single species lifetime while all molecules are immobilized. The synthetic trace generated using a lifetime of $\tau = 1\ ns$. The blue dots represent single photon arrival times (y-axis) recorded after each excitation pulse (x-axis). We consider the excitation pulse as a Gaussian IRF (Eq. 4) occurs at a frequency of 40 MHz with standard deviation of 0.1 ns. (B1) In the analysis to determine lifetimes, we first start with just 50 photons, first black-dashed line in panel (A), and gradually increase the number of photons considered in the analysis to (B2) 100, second black-dashed line in panel (A), (B3) 500, third black-dashed line in panel (A), and (B4) 1000 photons, last black-dashed line in panel (A). The ground truth for the lifetime is known (as this is synthetic data) and it is shown by the red-dashed line.

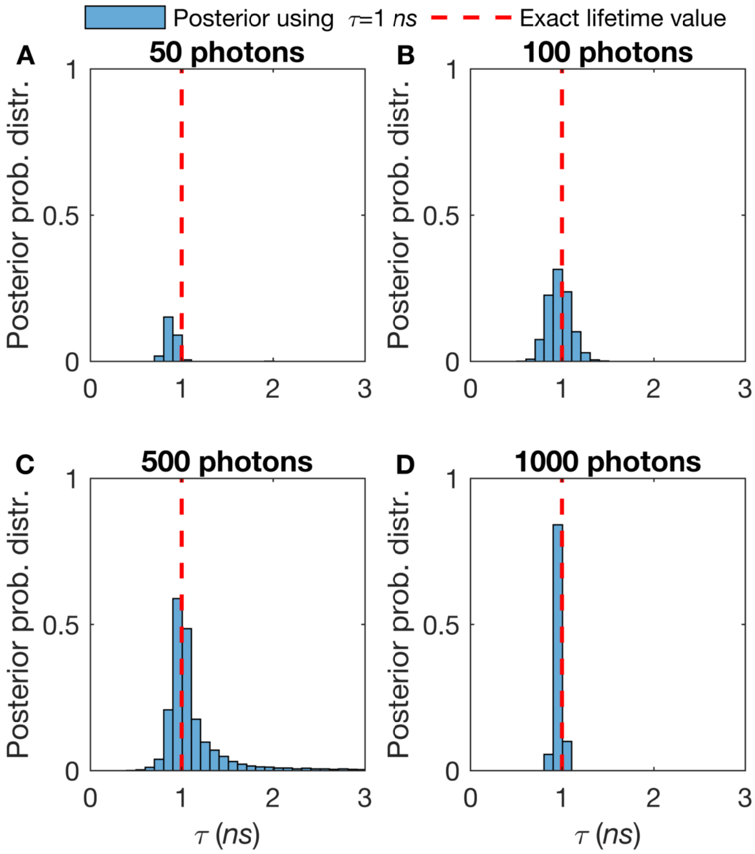

Figure S2: **Effect of the number of detected photons on a single diffusive molecular lifetime estimate. The more photons per unit time, the sharper the lifetime estimate.** Here, we work on single species lifetime while all molecules are diffusing with diffusion coefficient, $D = 10\ \mu m^2/s$. The synthetic trace is generated using $\tau = 1\ ns$. We start with 50 photons (A) and gradually increase the number of photons that we incorporate into the analysis to 100 (B), 500 (C), and 1000 (D) photons. The excitation pulses occur at a frequency of 40 MHz and we assume that these pulses assume a Gaussian shape with standard deviation of 0.1 ns. The ground truth for the lifetimes are known (as this is synthetic data) and they are shown by red-dashed lines.

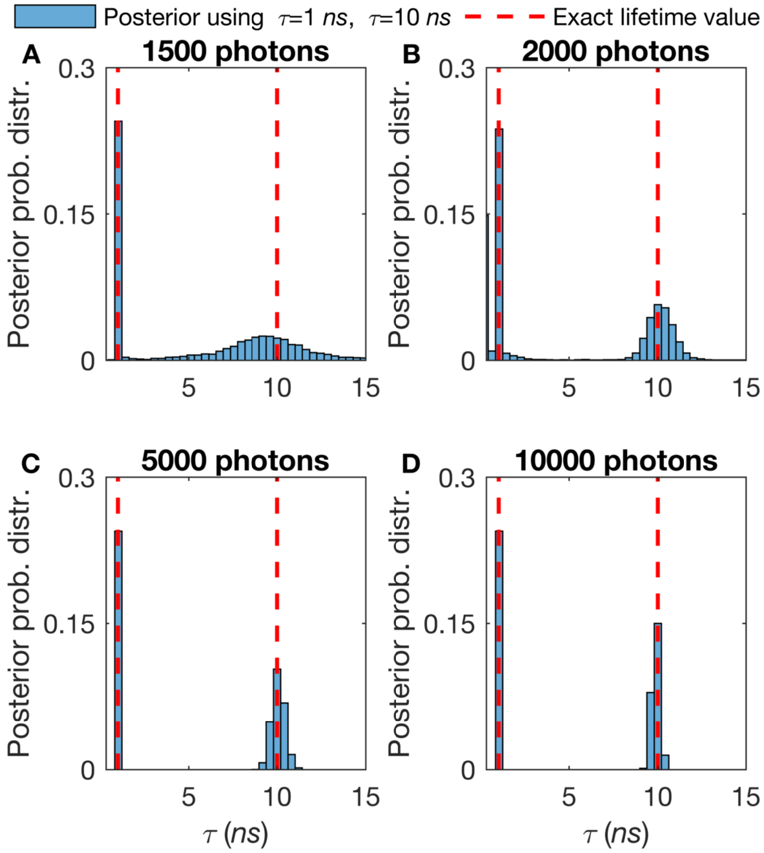

Figure S3: **Effect of the number of detected photons on a double diffusive molecular lifetime estimation. The more photons per unit time and thus the sharper estimation of lifetime.** Here, we work on single species lifetime while all molecules are diffusing with diffusion coefficient, $D = 10\ \mu m^2/s$. The synthetic trace generated by $\tau = 1\ ns$ and $\tau = 10\ ns$. We start with 1500 photons (A) and gradually increase the number of photons that we incorporate into the analysis to 2000 (B), 5000 (C), and 10000 (D) photons. Here, all other features such as the frequency of acquisition and width of pulse are the same as in Fig. S2. Also, we follow the same red-dashed line convention as in S1.

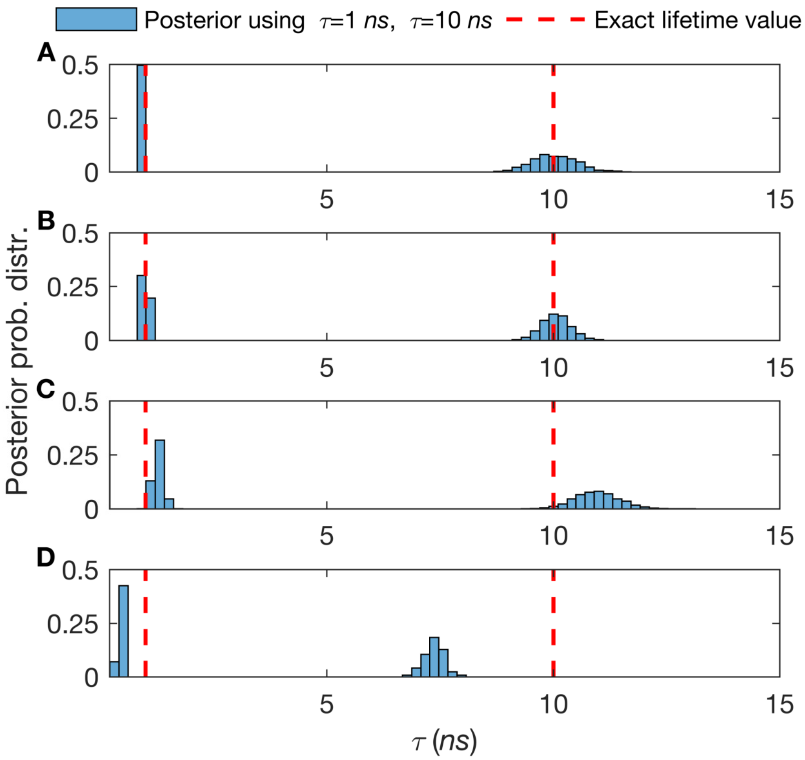

Figure S4: **Effect of the number of background photons on a double diffusive molecular lifetimes estimation. The more background photons per unit time, the poorer the lifetime estimate.** Here, we work on double species lifetime while all molecules are diffusing with diffusion coefficient, $D = 10\ \mu m^2/s$. The synthetic trace generated by $\tau = 1\ ns$ and $\tau = 10\ ns$ with total 3000 photons. We start with 3 background photons (A) and gradually increase the number of photons that we incorporate into the analysis to 30 (B), 150 (C), and 300 (D) photons. Here, all other features such as the frequency of acquisition and width of pulse are the same as in Fig. S2. Also, we follow the same red-dashed line convention as in S1.

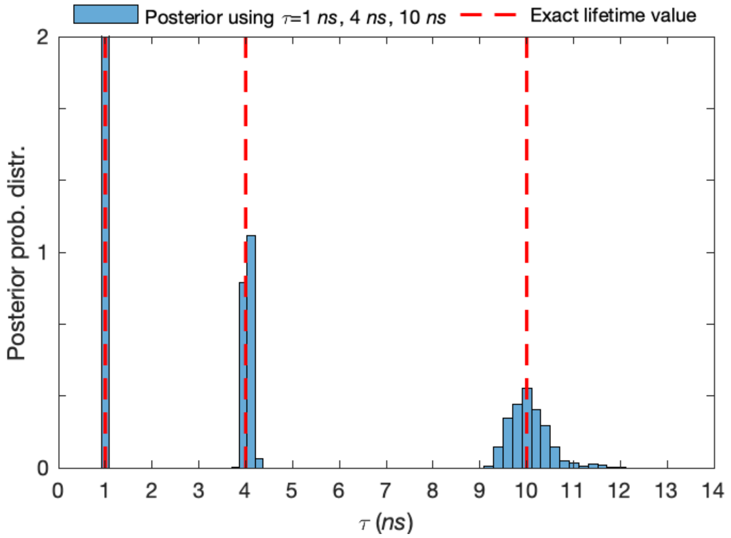

Figure S5: **Lifetime estimates with three different species using synthetic data.** Here, we generate a synthetic trace with three species having lifetimes $\tau = 1\ ns$, $\tau = 4\ ns$ and $\tau = 10\ ns$ with equal fraction of molecules contributing photons from different species (33% for each of them) and analyze a total of 2×10^5 photon arrivals. Here, all other features such as the frequency of acquisition and width of pulse are the same as in Fig. S2. Also, we follow the same red-dashed line convention as in S1.

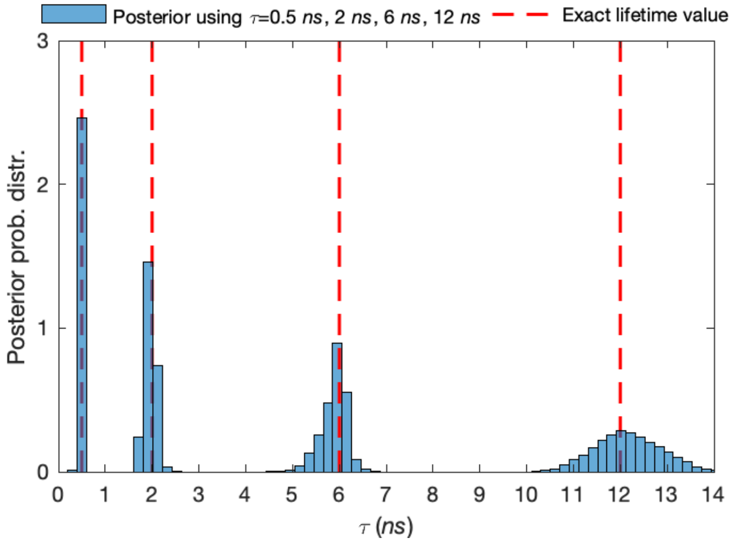

Figure S6: **Lifetime estimates with four different species in synthetic data.** Here, we work with four species lifetimes while all molecules are immobilized. The synthetic trace generated by $\tau = 0.5\ ns$, $\tau = 2\ ns$, $\tau = 6\ ns$ and $\tau = 12\ ns$ with equal fraction of molecules of each species (*i.e.*, set at 25%) for each of them and analyze a total of 3×10^5 photon arrivals. Here, all other features such as the frequency of acquisition and width of pulse are the same as in Fig. S2. Also, we follow the same red-dashed line convention as in S1.

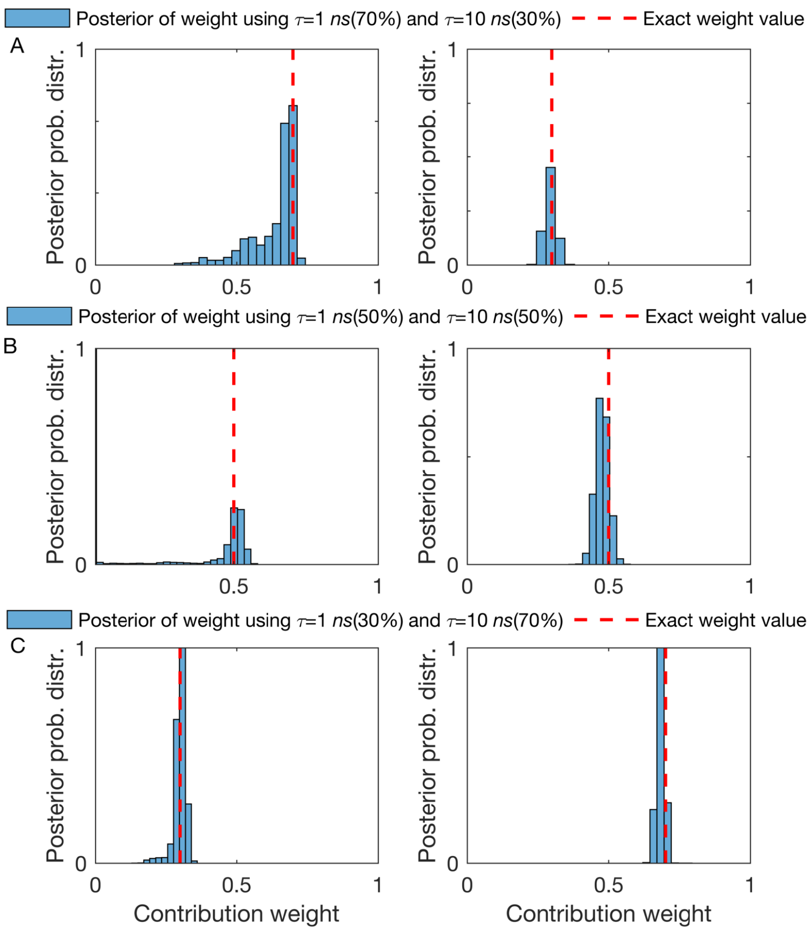

Figure S7: **Estimation of the fraction of molecules contributing photons from different species**. (A-C) Using the same synthetic traces as in Fig. 4, the posterior probability distribution over the fraction of molecules contributing photons from different species (weight) with lifetimes of 1 ns and 10 ns, 3000 total number of detected photons analyzed and fractions of chemical species of $70\% - 30\%$, $50\% - 50\%$ and $30\% - 70\%$ respectively. Here, all other features such as the frequency of acquisition and width of pulse are the same as in Fig. S2. Also, we follow the same red-dashed line convention as in S1.

Analysis of additional experimental data

Here, we used real measurements, obtained as explained in the method section, from different fluorescent dyes, namely Cy3, TMR, Rhod-B, and Rhod-6G. In Fig. S8 we considered a mixture of all four species. In Fig. S9 we show that we can correctly identify the fraction of molecules contributing photons from different species.

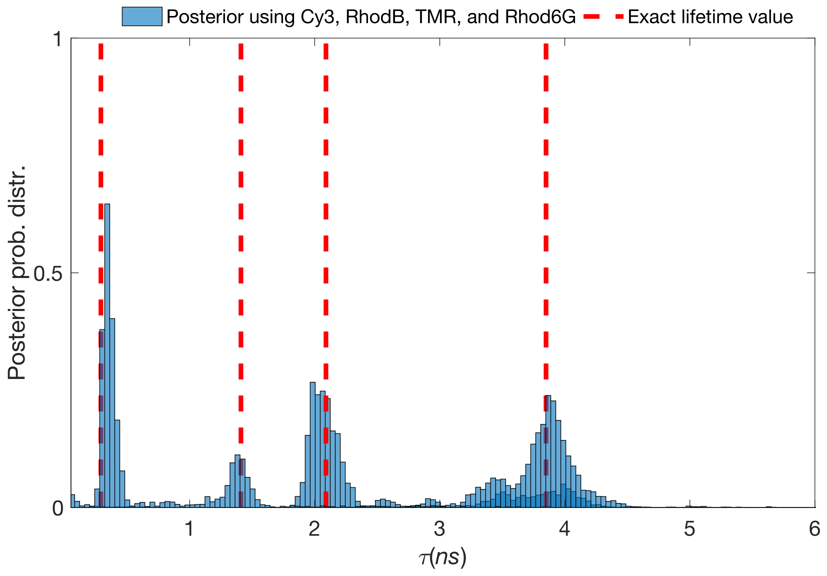

Figure S8: **Lifetime estimates for the case of four different species from experimental data.** Here, we work on four species lifetimes while all molecules are immobilized. The experimental trace generated by four different dyes including Cy3, Rhod-B, TMR, and Rhod-6G with a total of $\approx 3 \times 10^5$ photon arrivals analyzed. The excitation pulses occur with a frequency of 40 MHz and we assume that these pulses are modeled by a Gaussian with a standard deviation of 0.1 ns. The ground truth estimates for the lifetimes are determined using the whole trace which includes total 1.4×10^6 photon arrivals and they are shown by red-dashed lines.

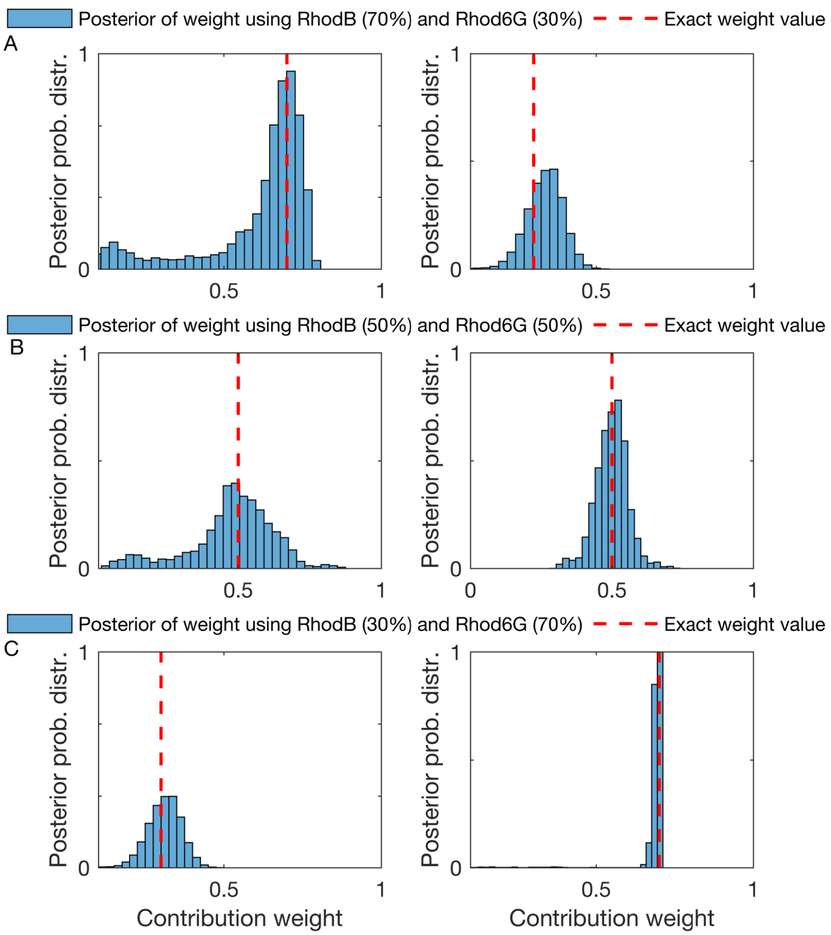

Figure S9: **Estimation of the different fraction of molecules contributing photons from different species from experimental data**. (A-C) Using the same traces as Fig. 8, the posterior probability distributions for the fraction of chemical species contributing photons for experimental dyes, RhodB and Rhod6G, with a total of ≈ 3000 total number of detected photons and fraction of chemical species contributing photons of $70\% - 30\%$, $50\% - 50\%$ and $30\% - 70\%$ respectively. The excitation pulses happen at a frequency of 40 MHz and we consider them to have a Gaussian shape with a standard deviation of 0.1 ns. What we treat as ground truth lifetime estimates (as we do not have real ground truths for experimental data) are determined using the whole trace which includes a total of 1.4×10^6 photon arrivals and they are shown by red-dashed lines.

Brief description of phasor plots analysis

Time domain

In typical time-domain lifetime imaging, a pulsed laser is used to excite the sample periodically, causing fluorescence emission for those pulses where a molecule is excited and decays back to the ground state radiatively. Experimentally, based on the data we presented, this is typically 1 in 40 pulses[2].

From Eq. 1, fluorescence species with M different lifetimes have exponentially decaying intensities

$$I(t) = \sum_{m=1}^{M} a_m \exp\left(-\frac{t}{\tau_m}\right) \tag{S1}$$

with fluorescence lifetimes τ_m and weights, a_m. In an ideal scenario, a fluorophore is excited with an exceedingly thin (Dirac-shaped) laser pulse at time $t = 0$. Its initial intensity is therefore $I(t < 0) = 0$. As excitation pulses are not infinitely sharp and detectors exhibit delays, the recorded signal, $\hat{I}(t)$, is the convolution of its fluorescence intensity $I(t)$ with the instrumental response function (IRF) [3, 4]; see Eq. S12.

Frequency Domain

Frequency-domain experiments constitute an alternative way to measure excited state lifetimes. In this case, the sample is excited with an intensity-modulated light, typically a sine-wave [2]. When a fluorescent sample is excited in this way, the emission intensity follows a shifted modulation (m) pattern with the phase shift (ϕ) and peak height that both encode information on the excited state lifetime [2].

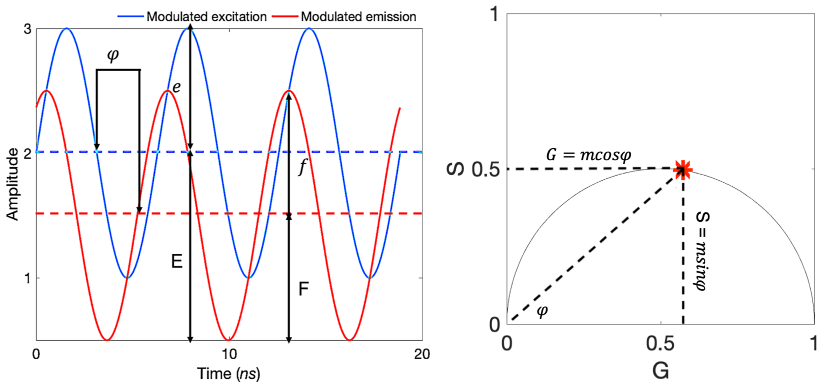

Figure S10: **Frequency-domain analysis.** Mapping frequency-domain modulated emission (left) into a phasor plot representation (right).

The modulation of the excitation is given by $\frac{e}{E}$, where e is the average intensity and E is the peak-to-peak height of the incident light (Fig. S10). The modulation of the emission is defined similarly, $\frac{f}{F}$, except using the intensities of the emission (Fig. S10). The shifted modulation between emission and excitation, $m = (f/F)/(e/E)$. The other experimental observable is the phase shift, (ϕ) which is the phase difference between excitation and emission. Both phase shift (ϕ) and the shifted modulation between emission and excitation (m) can be employed to calculate the lifetime using

$$\tan\phi = \omega\tau_\phi \tag{S2}$$

$$m = \frac{1}{\sqrt{1+\omega^2\tau_m^2}}. \tag{S3}$$

These expressions can be also be used to calculate the phase (τ_ϕ) and shifted modulation (τ_m) lifetimes for the curves shown in Fig. S10. If the intensity decay is a single exponential, then Eqs. S2 and S3 yield the correct lifetime. In this case, both τ_ϕ and τ_m are equal. For more than one species, these two are not the same and details are discussed in Ref. [2]

Along these same lines, lifetimes can also be determined using a phasor approach first introduced by Jameson et al. [5].

Briefly, we introduce the pair of conjugate variables G and S (termed phase coordinates) where

$$G = \frac{\int_0^{+\infty} I(t)\cos(\omega t)dt}{\int_0^{+\infty} I(t)dt} \qquad S = \frac{\int_0^{+\infty} I(t)\sin(\omega t)dt}{\int_0^{+\infty} I(t)dt} \tag{S4}$$

and where $I(t)$ is the photon intensity [6, 3]. In the case of single exponential $I(t) = a\exp(-\frac{t}{\tau})$, the coordinates of the phasor are given by

$$G = \frac{1}{1+(\omega\tau)^2} \qquad S = \frac{\omega\tau}{1+(\omega\tau)^2}. \tag{S5}$$

IRF approximation

To incorporate the effect of the IRF in our analysis, we approximate the IRF with a Gaussian function [7]; see Fig. S11. Centrally symmetric pulses such as the Gaussian, are obtained from electronics as used in most modern instruments [8]. However, non-symmetrical IRFs could be handled by proper modifications to Eq. 4 in the main text.

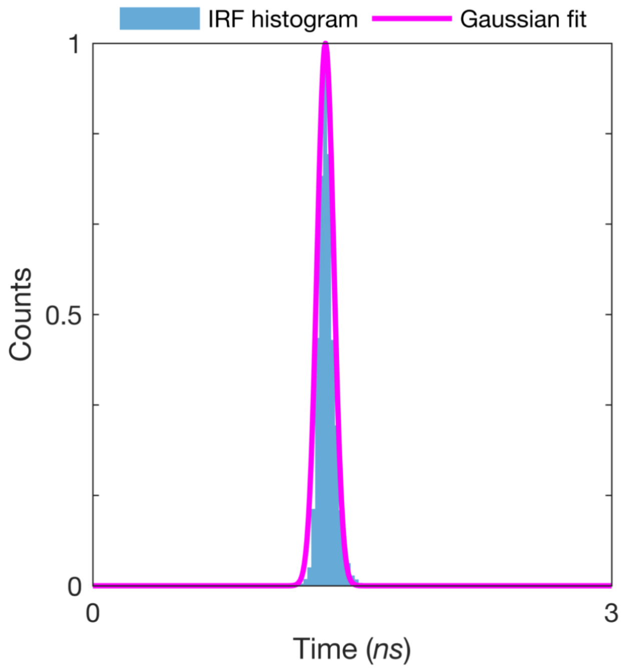

Figure S11: **The actual IRF (blue color) fitted with a Gaussian function (magenta color).** The fitted IRF is used for the analysis of all experimental data.

Description of the pulsed excitation and microtimes simulation

To simulate experimentally realistic microtimes, for mobile particles, we simulate diffusive molecules which freely traverse through an illuminated confocal volume. We define periodic boundaries [] ($\pm L_x$, $\pm L_y$, $\pm L_z$) which are much larger than the confocal radii to maintain a constant concentration of molecules. The confocal volume itself is pulsed on and off and the probability of excitation of a molecule depends on its location within that volume during the pulse. Here we consider the confocal volume (the combined excitation and emission point spread function, PSF) to be a 3D Gaussian, with radii of $\omega_x = 0.3$ μm, $\omega_y = 0.3$ μm, $\omega_z = 3.5$ μm and centered at the point of origin. The precise formula for this PSF is

$$\mathbf{PSF}\left(x, y, z\right) = \exp\left(-2\left(\left(\frac{x}{\omega_x}\right)^2 + \left(\frac{y}{\omega_y}\right)^2 + \left(\frac{z}{\omega_z}\right)^2\right)\right). \tag{S6}$$

So, the emission that received by molecule n of the mth is species

$$\mu_{m,n} = \mu_{m,ext}\mathbf{PSF}\left(x, y, z\right) \tag{S7}$$

where, $\mu_{m,ext}$ is the maximum excitation rate of the molecule n of species m which occurs when the molecule is at the center of the confocal volume [9].

Assuming that molecules do not move significantly over the duration of the pulse (of typical width 0.1 ns [10]), the probability of excitation of molecule n of species m is $q_{m,n} = \mu_{m,n}\delta t_p$ where, δt_p is the duration of the pulse. So, for any pulse excitation, we need to determine if the nth molecule of species m is excited or not. We define the variable $b_{m,n}$ to be either 1 or 0 if the molecule emits or does not emit a photon and consider this variable to be Bernoulli distributed

$$b_{m,n} \sim \mathbf{Bernoulli}\left(q_{m,n}\right). \tag{S8}$$

At the end, when a molecule is excited by each pulse $b_{m,n} = 1$, we need to consider the delays and errors introduced by the measuring electronic devices, $t_{det,k} - t_{ems,k}$. Since, we consider these errors follow a normal distribution, and the excitation time is normal distributed as well, we denote both effects with $\Delta t_{err,k} = (t_{ext,k} - t_{pul,k}) + (t_{det,k} - t_{ems,k})$ and as the result, we sample it from a normal distribution

$$\Delta t_{err,k} \sim \mathbf{Normal}(\tau_{IRF}, \sigma^2_{IRF}) \tag{S9}$$

where τ_{IRF} is the mean of IRF and σ_{IRF} is the standard deviation of the IRF (see Eq. 4 for comparison). In this simulation we considered $\sigma = \frac{\delta t_p}{2}$ as the width of the pulse.

After sampling the error time, we sample the emission time of each molecule from the exponential distribution with corresponding inverse lifetime belongs to species m

$$\Delta t_{ext,k}|\lambda_m \sim \mathbf{Exponential}\left(\lambda_m\right) \tag{S10}$$

and as we have shown in the Fig. S12 the detection time of each molecule will be sum of these two times

$$\Delta t_k = \Delta t_{ext,k} + \Delta t_{err,k} \tag{S11}$$

which is determined by the convolution of emission profile, Eq. S9, and excitation pulse, Eq. S10.

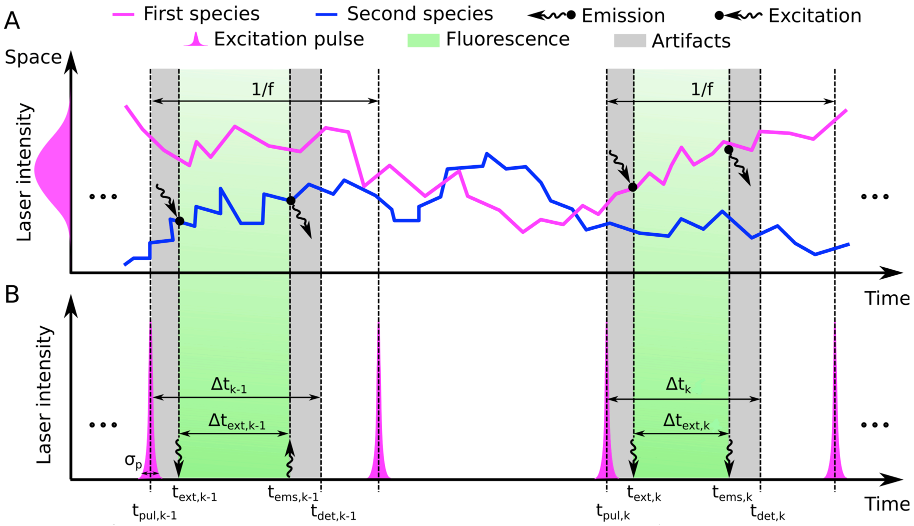

Figure S12: **Pictorial representation of the experimental setup a sample with a mixture of two species.** (A) The Brownian motion of two species in space versus time. Excitation and emission points are shown with different arrows. (B) Micro-times are the time between the peak of the pulse $t_{pul,k}$ that trigger the kth photon detection and detection time $t_{det,k}$. The time between the excitation $t_{ext,k}$ and emission $t_{ems,k}$ of the molecule, $\Delta t_{ext,k}$ follows the molecular lifetime. The gray and green-shaded regions are described in Fig. 9.

Derivation of model likelihood

As we mentioned in the main text, Section "Model description" , measurements $\Delta t_k = \Delta t_{ext,k} + \Delta t_{err,k}$, follow

$$\Delta t_k|\lambda_{s_k} \sim \mathbf{Normal}(\tau_{\text{IRF}}, \sigma^2_{\text{IRF}}) * \mathbf{Exponential}(\lambda_{s_k}). \tag{S12}$$

In this case we have

$$\begin{aligned}\Delta t_k|\lambda_{s_k} &\sim \int_{-\infty}^{\infty} \mathbf{Normal}(\tau_{\text{IRF}}, \sigma^2_{\text{IRF}})\mathbf{Exponential}(\lambda_{s_k}) d\Delta t_{ext} \\ &= \frac{\lambda_{s_k}}{\sqrt{2\pi\sigma^2_{\text{IRF}}}} \int_{-\infty}^{\infty} e^{-\frac{(\Delta t_k - \Delta t_{ext} - \tau_{\text{IRF}})^2}{2\sigma^2_{\text{IRF}}}} e^{\Delta t_{ext}\lambda_{s_k}} d\Delta t_{ext} \\ &= \frac{\lambda_{s_k}}{2} \exp\left[\frac{\lambda_{s_k}}{2}\left(2\left(\tau_{\text{IRF}} - \Delta t_k\right) + \lambda_{s_k}\sigma^2_{\text{IRF}}\right)\right] \text{erfc}\left(\frac{\tau_{\text{IRF}} - \Delta t_k + \lambda_{s_k}\sigma^2_{\text{IRF}}}{\sigma_{\text{IRF}}\sqrt{2}}\right)\end{aligned} \tag{S13}$$

where erfc($\cdot$) denotes the complementary error function.

Detailed description of the inference framework

Description of prior probability distributions

Within the Bayesian approach, all unknown model parameters need priors. The model parameters in our framework that require priors are: the inverse lifetimes $\{\lambda_m\}_m$; labels on each species $\overline{s}$; and probability on the labels of species $\overline{\pi}$ (fraction of molecules contributing photons from different species). Our choices of priors are described below.

Inverse lifetimes, $\{\lambda_m\}_m$. Here we are faced with different species which have different lifetimes. For convenience, we consider inverse lifetimes instead of lifetimes, $\tau_m = \frac{1}{\lambda_m}$, where the τ_m is the molecular lifetime and λ_m is the inverse lifetime of species m.

To learn inverse lifetimes, and to guarantee that their sampled values in our formulation attain only positive values, we place a Gamma distribution prior over them as follows

$$\lambda_m \sim \mathbf{Gamma}\left(\alpha_\lambda, \beta_\lambda\right), \tag{S14}$$

where, α_λ and β_λ are prior parameters.

Weights, $\overline{\pi}$. The weight on each species comes from the Dirichlet distribution

$$\overline{\pi} \sim \mathbf{Dirichlet}_M\left(\frac{\alpha}{M}, \dots, \frac{\alpha}{M}\right) \tag{S15}$$

where α is the scalar parameter of the Dirichlet distribution [11, 12]. This prior is conjugate to the labeled species, s_k, which simplifies the computations shown below. The Dirichlet distribution is an important multivariate continuous distribution in Bayesian statistics which is a multivariate generalization of the Beta distribution and, conveniently, conjugate to the Categorical [13].

Labels on each species, s_k

Since we have many species, we define a label for each molecule which will tell us that molecule belongs to which species

$$s_k|\overline{\pi} \sim \textbf{Categorical}_{1:M}\left(\overline{\pi}\right) \tag{S16}$$

where $\overline{\pi} = (\pi_1, \ldots, \pi_M)$ is the weight on each species. In other words, π_m is the fraction of photons which species m contributes to the data.

Summary of model equations

For concreteness, below we summarize all equations used in our framework, including a complete list of priors.

$$\lambda_m \sim \textbf{Gamma}\left(\alpha_\lambda, \beta_\lambda\right) \tag{S17}$$

$$\overline{\pi} \sim \textbf{Dirichlet}_M\left(\frac{\alpha}{M}, \ldots, \frac{\alpha}{M}\right) \tag{S18}$$

$$s_k|\overline{\pi} \sim \textbf{Categorical}_{1:M}\left(\overline{\pi}\right) \tag{S19}$$

$$\Delta t_k|\lambda_m, s_k \sim \frac{\lambda_{s_k}}{2}\exp\left[\frac{\lambda_{s_k}}{2}\left(2\left(\tau_{\mathrm{IRF}} - \Delta t_k\right) + \lambda_{s_k}\sigma^2_{\mathrm{IRF}}\right)\right]\mathrm{erfc}\left(\frac{\tau_{\mathrm{IRF}} - \Delta t_k + \lambda_{s_k}\sigma^2_{\mathrm{IRF}}}{\sigma_{\mathrm{IRF}}\sqrt{2}}\right) \tag{S20}$$

Inverse problem

Within the Bayesian paradigm, our goal is to sample from the following posterior probability distribution $\mathbb{P}\left(\{\lambda_m\}_m, \overline{s}, \overline{\pi}|\boldsymbol{\Delta t}\right)$. Since, it is not possible to directly compute this distribution, we will sample the random variables $\{\lambda_m\}_m$, $\overline{s}$, and $\overline{\pi}$ from their conditional distributions through a Gibbs sampling scheme [14, 15, 16, 17, 18]. Accordingly, posterior samples are generated by updating each one of the variables involved sequentially by sampling conditioned on all other variables and the measurements $\boldsymbol{\Delta t}$.

Conceptually, the steps involved in the generation of each posterior sample $(\{\lambda_m\}_m, \overline{s}, \overline{\pi})$ are:
Update the weights on each species $\overline{\pi}$
Update the labels on species $\overline{s}$
Update the inverse lifetimes $\{\lambda_m\}_m$.

Sampling of the weights $\overline{\pi}$. To update the weights of the labels on the species $\overline{s}$, we sample them from the corresponding conditional probability $\mathbb{P}\left(\overline{\pi}|\{\lambda_m\}_m, \boldsymbol{\Delta t}, \overline{s}, \right)$,

which simplifies to $\mathbb{P}(\overline{\pi}|\overline{s})$.

$$\begin{aligned}\overline{\pi} &\sim \mathbb{P}(\overline{\pi}|\overline{s}) \propto \mathbb{P}(\overline{s}|\overline{\pi})\,\mathbb{P}(\overline{\pi})\\ &= \left[\prod_{k=1}^{K}\mathbb{P}(s_k|\overline{\pi})\right]\mathbb{P}(\overline{\pi}) = \left[\prod_{k=1}^{K}\pi_{s_k}\right]\mathbf{Dirichlet}_M\left(\frac{\alpha}{M},\ldots,\frac{\alpha}{M}\right)\\ &= \left[\prod_{k=1}^{K}\pi_{s_k}\right]\frac{\Gamma\left(\sum_{m=1}^{M}\frac{\alpha}{M}\right)}{\sum_{m=1}^{M}\Gamma\left(\frac{\alpha}{M}\right)}\prod_{m=1}^{M}\pi_m^{\frac{\alpha}{M}-1}\\ &= \mathbf{Dirichlet}_M\left(\frac{\alpha}{M}+\sum_{k=1}^{K}\mathbb{I}(s_k=1),\ldots,\frac{\alpha}{M}+\sum_{k=1}^{K}\mathbb{I}(s_k=M)\right).\end{aligned}$$

Sampling of the labels $\overline{s}$. To sample the labels on species, we sample them from the conditional probability distribution $\mathbb{P}(s_k|\Delta t_k,\{\lambda_m\}_m,\overline{\pi})$ as follows

$$\begin{aligned}s_k &\sim \mathbb{P}(s_k|\Delta t_k,\{\lambda_m\}_m,\overline{\pi}) \propto \mathbb{P}(\Delta t_k|\{\lambda_m\}_m,s_k)\,\mathbb{P}(s_k|\overline{\pi})\\ &= \mathbf{Categorical}_{1:M}\Bigg(\pi_1\frac{\lambda_{s_k}}{2}\exp\left[\frac{\lambda_{s_k}}{2}\left(2\left(\tau_{\text{IRF}}-\Delta t_k\right)+\lambda_{s_k}\sigma_{\text{IRF}}^2\right)\right]\text{erfc}\left(\frac{\tau_{\text{IRF}}-\Delta t_k+\lambda_{s_k}\sigma_{\text{IRF}}^2}{\sigma_{\text{IRF}}\sqrt{2}}\right),\\ &\quad\vdots\\ &\quad,\pi_M\frac{\lambda_{s_k}}{2}\exp\left[\frac{\lambda_{s_k}}{2}\left(2\left(\tau_{\text{IRF}}-\Delta t_k\right)+\lambda_{s_k}\sigma_{\text{IRF}}^2\right)\right]\text{erfc}\left(\frac{\tau_{\text{IRF}}-\Delta t_k+\lambda_{s_k}\sigma_{\text{IRF}}^2}{\sigma_{\text{IRF}}\sqrt{2}}\right)\Bigg),\quad k=1,\ldots,K.\end{aligned}$$

Sampling the inverse lifetimes $\{\lambda_m\}_m$. To sample λ_m, we sample from the corresponding conditional probability distribution $\mathbb{P}(\{\lambda_m\}_m|\boldsymbol{\Delta t},\overline{s})$ as follows

$$\begin{aligned}\{\lambda_m\}_m &\sim \mathbb{P}(\{\lambda_m\}_m|\boldsymbol{\Delta t},\overline{s}) \propto \mathbb{P}(\boldsymbol{\Delta t}|\{\lambda_m\}_m,\overline{s})\left[\prod_{m=1}^{M}\mathbb{P}(\lambda_m)\right]\\ &= \left[\prod_{k=1}^{K}\frac{\lambda_{s_k}}{2}\exp\left[\frac{\lambda_{s_k}}{2}\left(2\left(\tau_{\text{IRF}}-\Delta t_k\right)+\lambda_{s_k}\sigma_{\text{IRF}}^2\right)\right]\text{erfc}\left(\frac{\tau_{\text{IRF}}-\Delta t_k+\lambda_{s_k}\sigma_{\text{IRF}}^2}{\sigma_{\text{IRF}}\sqrt{2}}\right)\right]\\ &\quad\times\left[\prod_{m=1}^{M}\mathbf{Gamma}(\lambda_m;\alpha_\lambda,\beta_\lambda)\right].\end{aligned} \tag{S21}$$

Since, there is no closed form to sample $\{\lambda_m\}_m$, we sample it using the Metropolis algorithm with the proposal

$$\lambda_m^{\text{prop}} \sim \mathbf{Gamma}\left(\alpha_{\lambda_m}^{\text{prop}},\frac{\lambda_m^{\text{old}}}{\alpha_{\lambda_m}^{\text{prop}}}\right),\qquad m=1,\ldots,M$$

where, the $\alpha_{\lambda_m}^{\text{prop}}$ is the parameter of the proposal distributions for the inverse lifetime. Then, the acceptance ratio is equal to

$$r_\lambda = \frac{\mathbb{P}(\{\lambda_m^{\text{prop}}\}_{\text{m}}|\boldsymbol{\Delta t},\overline{s})}{\mathbb{P}(\{\lambda_m^{\text{old}}\}_m|\boldsymbol{\Delta t},\overline{s})}\frac{\text{Proposal}\left(\{\lambda_m^{\text{old}}\}_m|\{\lambda_m^{\text{prop}}\}_m\right)}{\text{Proposal}\left(\{\lambda_m^{\text{prop}}\}_m|\{\lambda_m^{\text{old}}\}_m\right)}.$$

Also, to avoid numerical underflow, we work with the logarithm of the acceptance ratio

$$\log r_\lambda = \left[\sum_{k=1}^{K} \log\left(\frac{\lambda^{\text{prop}}_{s_\text{k}} - \lambda^{\text{old}}_{s_\text{k}}}{2}\right) + (\Delta t_\text{k} - \tau_\text{IRF})\left(\lambda^{\text{old}}_{s_k} - \lambda^{\text{prop}}_{s_k}\right) + \frac{\sigma^2}{2}\left({\lambda^2}^{\text{prop}}_{s_k} - {\lambda^2}^{\text{old}}_{s_k}\right)\right]$$
$$+ \log\left(\frac{\text{erfc}\left(\frac{\tau_\text{IRF} - \Delta t_k + \lambda^{\text{prop}}_{s_k}\sigma^2_\text{IRF}}{\sigma_\text{IRF}\sqrt{2}}\right)}{\text{erfc}\left(\frac{\tau_\text{IRF} - \Delta t_k + \lambda^{\text{old}}_{s_k}\sigma^2_\text{IRF}}{\sigma_\text{IRF}\sqrt{2}}\right)}\right)$$
$$+ \left[\sum_{m=1}^{M} \left(2\alpha^{\text{prop}}_{\lambda_m} - \alpha_\lambda\right)\log\left(\frac{\lambda^{\text{old}}_m}{\lambda^{\text{prop}}_m}\right) + \left(\frac{\lambda^{\text{old}}_m - \lambda^{\text{prop}}_m}{\beta_\lambda}\right) + \alpha^{\text{prop}}_{\lambda_m}\left(\frac{\lambda^{\text{prop}}_m}{\lambda^{\text{old}}_m} - \frac{\lambda^{\text{old}}_m}{\lambda^{\text{prop}}_m}\right)\right]. \tag{S22}$$

So, at the end we will accept or reject the proposal if

$$\log r_\lambda \geq 0 \Rightarrow \lambda^{\text{new}}_m = \lambda^{\text{prop}}_\text{m}, \qquad m = 1, \ldots, M$$
$$\log r_\lambda < 0 \Rightarrow \lambda^{\text{new}}_m = \lambda^{\text{old}}_m, \qquad m = 1, \ldots, M$$

Label switching correction of the molecular lifetimes. Label switching is a well-known feature of BNPs [19]. It arises when we are exploring complex posterior distributions by MCMC algorithms and the likelihood of the model is invariant to the relabelling of mixture components [20]. The issue of label switching appears because the likelihood is invariant under permutation of the indices. Under symmetric priors, the posteriors also reflects the likelihood's invariance with respect to index permutation. As a result, in any MCMC algorithm, labels of the components can permute multiple times between iterations of the sampler [21, 22]. Concretely, here, due to exchangeability of the molecular lifetimes, at any iteration (i) of the Gibbs sampling scheme, the corresponding lifetime of the species m might switch with the molecule's lifetime of the species m'. This label switching does not affect the joint posterior over all lifetimes.

To undo such label switching, at any iteration of the Gibbs sampling we compare the sampled lifetimes $\{\tau^{(i)}_m\}_m$ and their weights $\{\pi^{(i)}_m\}_m$ with a fixed set of lifetimes $\{\tau^*_m\}_m$ and weights $\{\pi^*_m\}_m$. Based on the distances of the lifetimes at iteration (i) from the fixed set of lifetimes, which we chose, we correct for label switching. The simple choice for this distance can be the distance between the lifetimes, but, since label switching happens in the sampled lifetimes, and subsequently the weights of each molecular lifetime, the particular distance we use incorporates the emission probability and the weights of each molecular lifetime

$$d_{m,m'} = \int_0^\infty dt \, \left|\pi_m \mathbf{Exp}\left(t;\tau_m\right) - \pi^*_m \mathbf{Exp}\left(t;\tau^*_m\right)\right| \tag{S23}$$

and we solve the assignment problem is minimizing this distance over the species $\sum_{m=1}^{M} d_{m,m'}$. This problem and its computation can be done efficiently by applying the Hungarian algorithm [23, 24, 25].

Table S1: Probability distributions used and their densities. Here, the corresponding random variables are denoted by x.

Distribution	Notation	Probability density function	Mean	Variance
Normal	Normal(μ, σ^2)	$\frac{1}{\sqrt{2\pi\sigma^2}} e^{-\frac{(x-\mu)^2}{2\sigma^2}}$	μ	σ^2
Exponential	Exponential(μ)	$\mu e^{-\mu x}$	$\frac{1}{\mu}$	$\frac{1}{\mu^2}$
Gamma	Gamma(α, β)	$\frac{1}{\Gamma(\alpha)\beta^\alpha} x^{\alpha-1} e^{-\frac{x}{\beta}}$	$\alpha\beta$	$\alpha\beta^2$

Table S2: Here, we list point estimates of our analyses for synthetic data, which we obtain from the marginal posterior probability distributions $p(\tau|\boldsymbol{\Delta t})$. Estimates are listed according to figure.

	τ	
	mean	std
	ns	ns
Fig. 2C	0.51 , 2.19, 10.51	0.14 , 1.42 , 6.45
Fig. 2D	0.52 , 2.36 , 13. 01	0.26 , 1.65 , 12.59
Fig. 2E	0.52 , 2.51 , 9.74	0.31 , 2.33 , 15.74
Fig. 2F	0.51 , 2.10 , 11.06	0.32 , 0.65 , 6.71
Fig. S1B1	1.17	0.29
Fig. S1B2	1.03	0.23
Fig. S1B3	1.04	0.05
Fig. S1B4	1.01	0.03
Fig. 3B1	0.82 , 8.88	0.41 , 10.31
Fig. 3B2	1.10 , 10.37	0.33 , 6.31
Fig. 3B3	1.07 , 10.08	0.15 , 4.98
Fig. 3B4	1.01 , 10.1	0.05 , 5.23
Fig. 4A	0.95 , 9.21	0.21 , 8.91
Fig. 4B	1.10 , 10.13	0.35 , 7.11
Fig. 4C	1.07 , 10.08	0.15 , 10.18
Fig. 5A	1.05 , 10.12	0.14 , 3.84
Fig. 5B	1.10 , 5.11	0.25 , 3.11
Fig. 5C	0.87 , 2.18	0.98 , 2.06
Fig. 5D	1.13 , 1.48	0.26 , 0.68
Fig. S2A	0.85	0.31
Fig. S2B	1.03	0.39
Fig. S2C	0.99	0.48
Fig. S2D	1.01	0.11
Fig. S5	1.01 , 4.10 , 10.06	0.12 , 0.35 , 5.21
Fig. S6	0.51 , 1.97 , 6.16 , 12.25	0.14 , 0.55 , 3.41 , 7.43

Table S3: Here, we list point estimates of our analyses for experimental data, which we obtain from the marginal posterior probability distributions $p(\tau|\boldsymbol{\Delta t})$. Estimates are listed according to figure.

	τ	
	mean	std
	ns	ns
Fig. 6A1	3.14	2.49
Fig. 6B1	3.84	1.84
Fig. 6C1	3.85	0.37
Fig. 7A1	1.44 , 3.39	1.14 , 1.52
Fig. 7B1	1.42 , 3.86	0.46 , 1.05
Fig. 7C1	1.41 , 3.71	0.30 , 1.10
Fig. 8A1	1.44 , 3.42	0.48 , 1.62
Fig. 8B1	1.42 , 3.91	0.39 , 1.24
Fig. 8C1	1.37 , 3.75	1.12 , 1.15
Fig. S8	0.21 , 1.37 , 2.06 , 3.89	0.25 , 0.72 , 1.41 , 2.44

Supplemental Reference

1. Sun Y, Phipps J, Elson DS, Stoy H, Tinling S, Meier J, Poirier B, Chuang FS, Farwell DG, Marcu L. Fluorescence lifetime imaging microscopy: in vivo application to diagnosis of oral carcinoma. *Optics Letters* 2009;34(13):2081–3.

2. Lakowicz JR. Principles of fluorescence spectroscopy. Springer Science & Business Media; 2013.

3. Lakner PH, Monaghan MG, Möller Y, Olayioye MA, Schenke-Layland K. Applying phasor approach analysis of multiphoton flim measurements to probe the metabolic activity of three-dimensional in vitro cell culture models. *Scientific Reports* 2017;7:42730.

4. Warren SC, Margineanu A, Alibhai D, Kelly DJ, Talbot C, Alexandrov Y, Munro I, Katan M, Dunsby C, French PM. Rapid global fitting of large fluorescence lifetime imaging microscopy datasets. *PLoS One* 2013;8:e70687.

5. Jameson DM, Gratton E, Hall RD. The measurement and analysis of heterogeneous emissions by multifrequency phase and modulation fluorometry. *Applied spectroscopy reviews* 1984;20(1):55–106.

6. Digman MA, Caiolfa VR, Zamai M, Gratton E. The phasor approach to fluorescence lifetime imaging analysis. *Biophysical Journal* 2008;94(2):L14–6.

7. Rowley MI, Barber PR, Coolen AC, Vojnovic B. Bayesian analysis of fluorescence lifetime imaging data. In: *Multiphoton Microscopy in the Biomedical Sciences XI*; vol. 7903. International Society for Optics and Photonics; 2011:790325.

8. Rowley MI, Coolen AC, Vojnovic B, Barber PR. Robust Bayesian fluorescence lifetime estimation, decay model selection and instrument response determination for low-intensity FLIM imaging. *PloS One* 2016;11(6):e0158404.

9. Enderlein J, Ambrose WP. Optical collection efficiency function in single-molecule detection experiments. *Applied Optics* 1997;36(22):5298–302.

10. Otosu T, Ishii K, Oikawa H, Arai M, Takahashi S, Tahara T. Highly heterogeneous nature of the native and unfolded states of the B domain of protein a revealed by two-dimensional fluorescence lifetime correlation spectroscopy. *The Journal of Physical Chemistry B* 2017;121(22):5463–73.

11. Ferguson TS. A Bayesian analysis of some nonparametric problems. *The Annals of Statistics* 1973;:209–30.

12. Ng KW, Tian GL, Tang ML. Dirichlet and related distributions: Theory, methods and applications; vol. 888. John Wiley & Sons; 2011.

13. Lin J. On the dirichlet distribution. Ph.D. thesis; Masters thesis, Department of Mathematics and Statistics, Queens University ; 2016.

14. Gelman A, Carlin JB, Stern HS, Dunson DB, Vehtari A, Rubin DB. Bayesian data analysis; vol. 2. CRC press Boca Raton, FL; 2014.

15. Von Toussaint U. Bayesian inference in physics. *Reviews of Modern Physics* 2011;83(3):943.

16. Tavakoli M, Taylor JN, Li CB, Komatsuzaki T, Pressé S. Single molecule data analysis: An introduction. *arXiv preprint arXiv:160600403* 2016;.

17. Robert C, Casella G. Introducing Monte Carlo Methods with R. Springer Science & Business Media; 2009.

18. Liu H, Motoda H. Computational methods of feature selection. CRC Press; 2007.

19. Jasra A, Holmes CC, Stephens DA. Markov chain monte carlo methods and the label switching problem in Bayesian mixture modeling. *Statistical Science* 2005;:50–67.

20. Egidi L, Pappada R, Pauli F, Torelli N. Relabelling in Bayesian mixture models by pivotal units. *Statistics and Computing* 2018;28(4):957–69.

21. Stephens M. Dealing with label switching in mixture models. *Journal of the Royal Statistical Society: Series B (Statistical Methodology)* 2000;62(4):795–809.

22. Rodriguez CE, Walker SG. Label switching in bayesian mixture models: Deterministic relabeling strategies. *Journal of Computational and Graphical Statistics* 2014;23(1):25–45.

23. Mills-Tettey GA, Stentz A, Dias MB. The dynamic hungarian algorithm for the assignment problem with changing costs. *Technical Report CMU-RI-TR-07-27* 2007;:1–13.

24. Kuhn HW. The hungarian method for the assignment problem. *Naval Research Logistics Quarterly* 1955;2(1-2):83–97.

25. Munkres J. Algorithms for the assignment and transportation problems. *Journal of the Society for Industrial and Applied Mathematics* 1957;5(1):32–8.
